# Efficacy of Therapies for Solar Urticaria: A Systematic Review and Meta-Analysis

**DOI:** 10.3390/jcm14165736

**Published:** 2025-08-13

**Authors:** Maya Engler Markowitz, Yehonatan Noyman, Israel Khanimov, Itay Zahavi, Batya Davidovici, Riad Kassem, Daniel Mimouni, Assi Levi

**Affiliations:** 1 Division of Dermatology, Rabin Medical Center, Petach Tikva 49100, Israel; jnoyman@gmail.com (Y.N.); israel.khanimov@gmail.com (I.K.); bdavidovici@yahoo.com (B.D.); docalevi@gmail.com (A.L.); 2 Gray Faculty of Medical and Health Science, Tel Aviv University, Tel Aviv 69978, Israel; itai1994@gmail.com (I.Z.); riadkassem@hotmail.com (R.K.); 3 Department of Dermatology, Sheba Medical Center, Tel Hashomer, Ramt Gan 52621, Israel

**Keywords:** solar urticaria, photodermatoses, systematic review, meta-analysis, antihistamines, phototherapy, omalizumab

## Abstract

**Background**: Solar urticaria is a rare and disabling photodermatosis. Due to its low prevalence, most available data regarding treatment are derived from observational studies and case series, and a systematic evaluation of treatment efficacy is lacking. This systematic review and meta-analysis aims to assess therapeutic outcomes across treatment modalities in order to guide clinical care. **Methods**: We conducted a systematic literature search across PubMed, ScienceDirect, the Cochrane Library, and ClinicalTrials.gov. Studies reporting treatment outcomes in patients with solar urticaria were included. Pooled response rates were calculated for each treatment modality. **Results**: Out of 508 studies initially identified, 38 met the inclusion criteria. Antihistamines were evaluated in 21 studies (376 patients), with a pooled response rate (partial or complete) of 83.0% (95% CI, 70.4–91.1%) and a complete response rate of 7.7% (95% CI, 1.7–28.3%). Phototherapy was assessed in 11 studies (145 patients), showing a similar overall response (89.8%; 95% CI, 77.9–95.3%) but a higher complete response rate (39.8%; 95% CI, 18.3–66.1%). Omalizumab, evaluated in nine studies (76 patients), demonstrated the highest efficacy, with 93.2% (95% CI, 73.8–98.5%) achieving response and 68.4% (95% CI, 48.5–83.2%) complete remission. Limited data on IVIG, cyclosporine, and plasmapheresis suggested partial efficacy in selected refractory cases. **Conclusions**: This meta-analysis may support clinical decision-making by clinicians. A stepwise approach is suggested: high-dose H_1_ antihistamines as first-line therapy, phototherapy as an alternative option in patients with access to treatment centers, and omalizumab for those with insufficient response. In refractory cases, additional options might be considered.

## 1. Introduction

Solar urticaria is a rare and debilitating photodermatosis classified as a subtype of inducible (physical) urticaria. It is characterized by the rapid onset of pruritic erythema and wheals within minutes of exposure to light [1]. This reaction significantly impairs quality of life and can severely limit daily activities [2]. In affected individuals, symptoms are reproducibly triggered by specific wavelengths of radiation, most commonly within the ultraviolet A (UVA) and visible light (VL) spectra, as determined by phototesting [1].

Due to the limited effectiveness of sunscreens in protecting against UVA and VL [3], management of solar urticaria includes non-sedating H_1_ antihistamines as first-line treatment [4], with leukotriene receptor antagonists (LRAs) added in selected cases [5]. In patients with persistent symptoms, phototherapy using UVA, broadband UVB, or narrowband UVB (NB-UVB) may be attempted based partly on the individual’s action spectrum [6,7]. For refractory cases, omalizumab, an anti-IgE monoclonal antibody, is used [8]. Additional immunomodulatory treatments—including cyclosporine, plasmapheresis, and intravenous immunoglobulin (IVIG)—have been employed in isolated, treatment-resistant cases [9,10,11].

Due to the rarity of this condition, the existing body of evidence regarding therapeutic interventions is limited primarily to observational studies and small case series, with no large-scale studies systematically evaluating treatment outcomes. As a result, no clear treatment hierarchy has been established, and clinicians must often rely on limited data and anecdotal experience when making therapeutic decisions.

This systematic review and proportional meta-analysis aims to address this gap by evaluating the efficacy of various treatment modalities for solar urticaria. We aim to provide clinicians and patients with a clearer understanding of the relative effectiveness of current therapeutic strategies, support evidence-based decision-making, and highlight areas where further research is needed.

## 2. Materials and Methods

This systematic review and meta-analysis was conducted in accordance with the Preferred Reporting Items for Systematic Reviews and Meta-Analyses (PRISMA) guidelines [12]. The study was registered with the International Prospective Register of Systematic Reviews (PROSPERO; registration number: CRD420251063215).

### 2.1. Search Strategy

A comprehensive literature search was performed in MEDLINE (via PubMed), the Cochrane Library, ScienceDirect, and ClinicalTrials.gov to identify relevant studies evaluating treatment outcomes in patients with solar urticaria. Given the rarity of the condition, we used a broad and inclusive search strategy based solely on the term “solar urticaria”, without restricting the search by treatment type, in order to maximize sensitivity and avoid omitting relevant studies.

The literature search was conducted in May 2025 and included all records available from the inception of each database through the search date. Only full-text articles published in peer-reviewed journals and written in English were considered. Two reviewers (MEM and IK) independently screened all titles and abstracts, assessed full texts for eligibility, and extracted data. Disagreements were resolved through discussion and consensus; if consensus could not be reached, a third reviewer (YN) was consulted. In addition, the reference lists of included studies were manually reviewed to identify any additional eligible publications.

#### 2.1.1. Inclusion Criteria

We included studies of any design, including observational (retrospective or prospective) studies, case series, and analytical studies (e.g., comparative cohort studies and randomized controlled trials), that met all of the following criteria: (a) reported on patients with a clinical diagnosis of solar urticaria, regardless of age, sex, or disease severity; (b) included at least 3 patients with solar urticaria; (c) provided data on any type of treatment modality used (pharmacologic, phototherapeutic, etc.); and (d) reported treatment response, assessed either through clinical evaluation or via phototesting.

#### 2.1.2. Exclusion Criteria

Studies were excluded if they met any of the following criteria: (a) included fewer than 3 patients diagnosed with solar urticaria; (b) did not report the treatment modality administered; (c) did not include any form of treatment response assessment, either clinical or by phototesting; (d) were not published in English; (e) were not available as full-text articles in peer-reviewed journals; or (f) reported duplicate patient populations, identified either by explicit statements within the manuscript or by overlapping demographic and clinical data with previously published cohorts.

### 2.2. Data Extraction

Data from all included studies were extracted using a standardized data collection form in Excel. For each study, we recorded the following information: study characteristics (first author, year of publication, country, and study design); patient characteristics (number of patients, age, sex, and disease severity, if reported); diagnostic criteria for solar urticaria; details of the treatment regimen (type of treatment, dosage, frequency, duration, and use of combination therapies); and outcomes, including treatment response (primary outcome), adverse effects (type and severity), and length of follow-up.

#### Definition and Classification of Primary Outcome

The primary outcome was response to treatment, classified as complete response, partial response, or no response. This categorization was based on the clinical assessment reported in each study. When studies did not explicitly define these categories, we derived the classification from the descriptive clinical information provided. In cases of ambiguity, responses were conservatively categorized as less favorable (e.g., partial rather than complete, or no response rather than partial).

For studies that assessed response exclusively through phototesting, classification was performed using a predefined scale: a mean urticaria dose (MUD) increase of ≥10-fold or normalization was defined as a complete response, a 1.1- to 9.9-fold increase as a partial response, and a <1.1-fold increase as no response.

All treatment responses were independently assessed by two reviewers (MEM and IK). Discrepancies in classification were resolved by discussion, and when consensus could not be reached, a third reviewer (YN) was consulted.

### 2.3. Quality Assessment

The methodological quality and risk of bias of the included studies were assessed using the Joanna Briggs Institute (JBI) Critical Appraisal Tools, which provide study-design-specific checklists tailored for different types of research designs. Case series were evaluated using the JBI Critical Appraisal Checklist for Case Series [13]. Cohort studies were assessed using the JBI Critical Appraisal Checklist for Cohort Studies. Case–control studies were assessed using the JBI Critical Appraisal Checklist for Case–Control Studies. Interventional (quasi-experimental) studies were evaluated using the JBI Critical Appraisal Checklist for Quasi-Experimental Studies (non-randomized) [14]. Randomized controlled trials (RCTs) were assessed using the JBI Critical Appraisal Checklist for Randomized Controlled Trials [15].

### 2.4. Data Analysis and Synthesis

Meta-analyses were performed separately for each treatment modality when at least 3 eligible studies were available (e.g., antihistamines, phototherapy, and omalizumab). Treatment modalities represented by fewer than 3 studies were summarized descriptively. Sensitivity analyses were conducted based on study design, with separate analyses for case series and for all other study types (excluding case series).

All analyses were performed in R (version 4.4.2), applying the lme4 package for generalized linear mixed model (GLMM) estimation and the meta package for forest-plot visualization. The random study effect was used to measure between-study heterogeneity using τ^2^ and I^2^ statistics. Ninety-five percent confidence intervals (95% CI) were calculated for all pooled estimates.

## 3. Results

### 3.1. Study Selection

**Figure 1:**
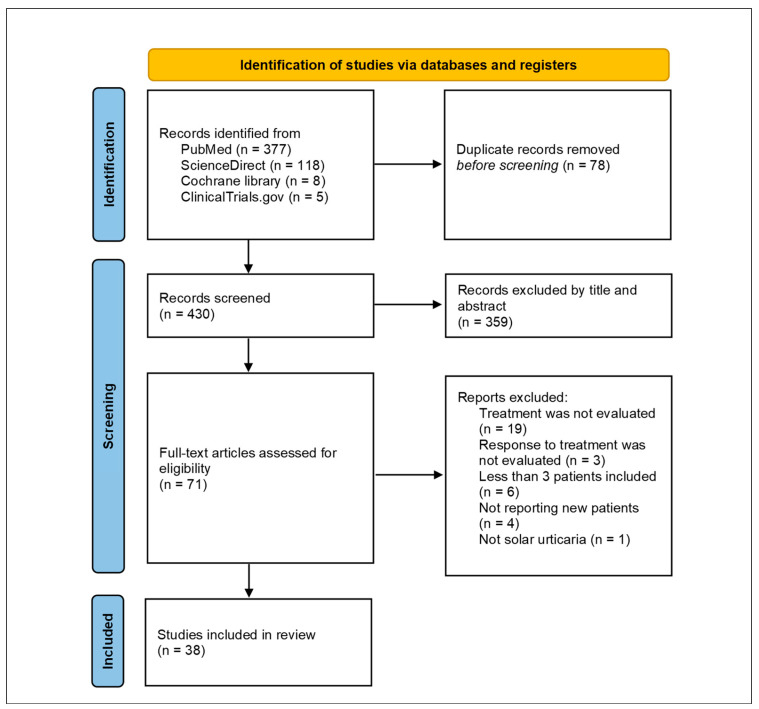
PRISMA flow diagram illustrating the literature search and selection process.

A total of 508 studies were identified through the initial database search. After removing duplicates and screening titles and abstracts, 71 full-text articles were assessed for eligibility. Of these, 38 studies met the inclusion criteria and were included in the final analysis. Reasons for exclusion are detailed in the PRISMA flow diagram (Figure 1).

The main characteristics of the included studies are summarized in Table 1. Among the included studies, 24 were case series [4,5,6,16,17,18,19,20,21,22,23,24,25,26,27,28,29,30,31,32,33,34,35,36], 10 were open-label interventional studies [7,37,38,39,40,41,42,43,44,45], 2 were randomized controlled trials [46,47], 1 was a case–control study [48], and 1 was a cohort study [49]. The methodological quality assessment of the studies is presented in the Supplementary Materials (Figure S1a–d).

### 3.2. Treatment Response Evaluation

Given the substantial variability in treatment response definition across studies, we summarized the assessment methods used in each report based on clinical evaluation, phototesting, both, or neither (not specified). Table 2 presents the distribution of studies and patients by type of response assessment. A more detailed breakdown by study is provided in the Supplementary Materials (Table S1).

#### 3.2.1. Response to Antihistamines

We analyzed 21 studies comprising 376 patients to assess the clinical response to antihistamine treatment [4,5,16,17,18,19,20,21,22,23,24,25,26,27,37,38,39,46,47,48,49].

In 10 studies [4,16,17,19,21,22,25,47,48,49], the specific antihistamine agents or dosages were not reported. In the remaining studies [5,18,20,23,24,26,27,37,38,39,46], treatment typically included H_1_ antihistamines, such as cetirizine, fexofenadine, loratadine, desloratadine, bilastine, ebastine, astemizole, and terfenadine. In several studies, combined regimens were used, involving multiple H_1_ antihistamines or the addition of an H_2_ antihistamine (e.g., cimetidine or ranitidine). Dosing regimens ranged from standard doses to up-dosing up to six times the usual dose, with variability both between studies and within individual studies across patients. In three studies, patients received a combination of antihistamines and LRAs [5,24,27].

**Figure 2:**
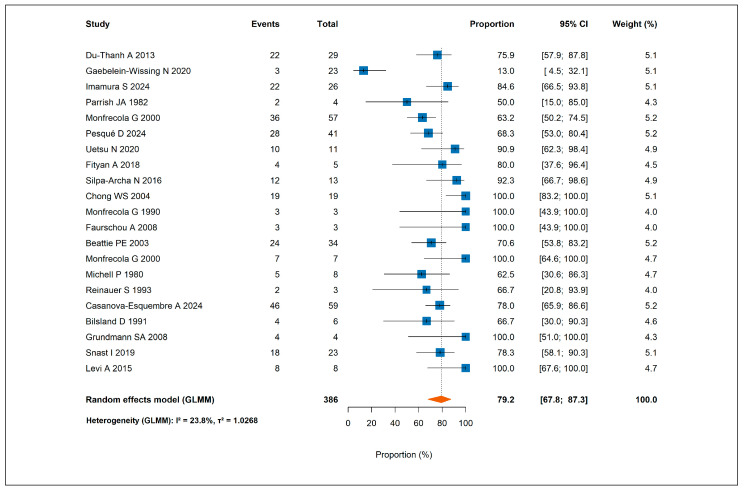
Forest plot showing the pooled rate of at least partial response to antihistamines across all included studies [4,5,16,17,18,19,20,21,22,23,24,25,26,27,37,38,39,46,47,48,49].

An at least partial response was observed in 282 patients, corresponding to a pooled response rate of 79.2% (95% CI, 67.8–87.3%) (Figure 2). A complete response was achieved in only 67 patients, with a pooled complete response rate of 7.6% (95% CI, 1.8–27.5%) (Supplementary Materials, Figure S2).

In a subgroup analysis of patients treated with antihistamines alone (without LRAs), pooled response rates for both partial and complete responses were similar to the overall estimates (Supplementary Materials, Figure S3a,b). In contrast, the separate analysis including only the three studies in which patients received both antihistamines and LRAs showed a comparable rate of at least partial response (87.2%; 95% CI, 37.8–98.7%), but a markedly higher rate of complete response (74.3%; 95% CI, 57.5–86.0%) (Supplementary Materials, Figure S4a,b).

Sensitivity analyses by study design revealed no substantial differences in response rates between subgroups (Supplementary Materials, Figure S5a–d).

Data on adverse events were explicitly reported in only three studies. Two studies noted no adverse events [23,27], while one study reported mild fatigue and drowsiness in some patients, which resolved spontaneously [5].

#### 3.2.2. Response to Phototherapy

We analyzed 11 studies comprising 145 patients to assess the clinical response to phototherapy [6,7,16,17,18,28,29,37,40,41,42]. Of these, nine studies (95 patients) evaluated UVA-based treatments [6,7,16,17,18,28,37,41,42], including three studies (52 patients) utilizing psoralen plus UVA (PUVA) [7,18,37] and three studies (32 patients) implementing UVA rush hardening protocols [6,16,17]. Two studies (47 patients) assessed the efficacy of UVB phototherapy [29,40]. One of the studies evaluating UVA treatment also included a separate treatment arm of three patients who received VL therapy [17]. These patients were excluded from the pooled analysis due to the distinct nature of this modality, and their clinical outcomes are described narratively.

Treatment protocols varied across studies. In most reports, phototherapy was administered two to three times per week over several weeks, with some studies implementing maintenance therapy during spring and summer months, while others did not specify maintenance regimens or duration of clinical response. Several studies advised patients to continue sun exposure during summer, typically two to three times per week in the early afternoon for 20 to 60 min, to help maintain tolerance.

**Figure 3:**
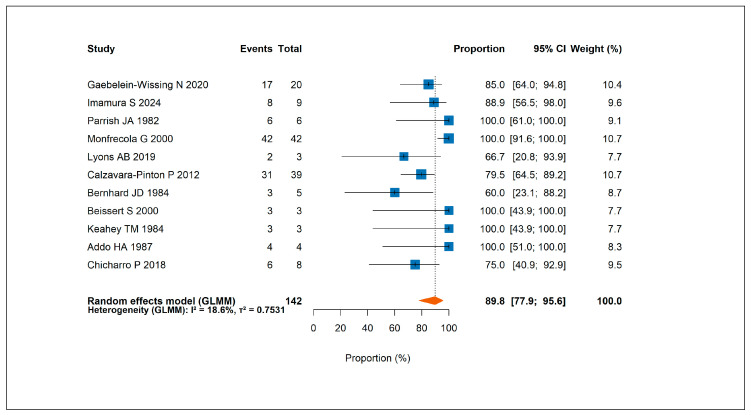
Forest plot showing the pooled rate of at least partial response to phototherapy across all included studies [6,7,16,17,18,28,29,37,40,41,42].

Across all phototherapy-treated patients, regardless of treatment modality, at least partial response was reported in 125 patients, corresponding to a pooled response rate of 89.8% (95% CI, 77.9–95.6%) (Figure 3). A complete response was achieved in 56 patients, with a pooled complete response rate of 39.8% (95% CI, 18.3–66.1%) (Supplementary Materials, Figure S6).

In a subgroup analysis stratified by phototherapy modality, overall response rates were comparable among patients treated with UVA-based therapies, including those receiving rush hardening protocols (Supplementary Materials, Figure S7), and those treated with non-rush UVA regimens (Supplementary Materials, Figure S8). Patients treated with UVB phototherapy exhibited slightly lower response rates; however, these remained within the broader confidence interval observed for the overall phototherapy cohort (Supplementary Materials, Figure S9).

Sensitivity analyses by study design revealed no substantial differences in response rates between subgroups (Supplementary Materials, Figure S10a–d). Notably, none of the three patients treated with visible light (VL) demonstrated a clinical response.

Data on adverse events were reported in five studies. One study noted no adverse events [29], while four others described various reactions. In two studies, only mild adverse events were noted, including urticaria, erythema, or pruritus [6,37]. The remaining two studies reported more significant reactions in individual patients, including a widespread flare response with associated panic and light-headedness [28] and urticarial streaks, dizziness, and throat swelling following higher UVA doses [7].

#### 3.2.3. Response to Omalizumab

We analyzed nine studies comprising 76 patients to assess the clinical response to omalizumab [16,27,30,31,32,35,43,48,49]. Across the included studies, omalizumab was administered every 2–4 weeks at doses ranging from 150 to 600 mg.

**Figure 4:**
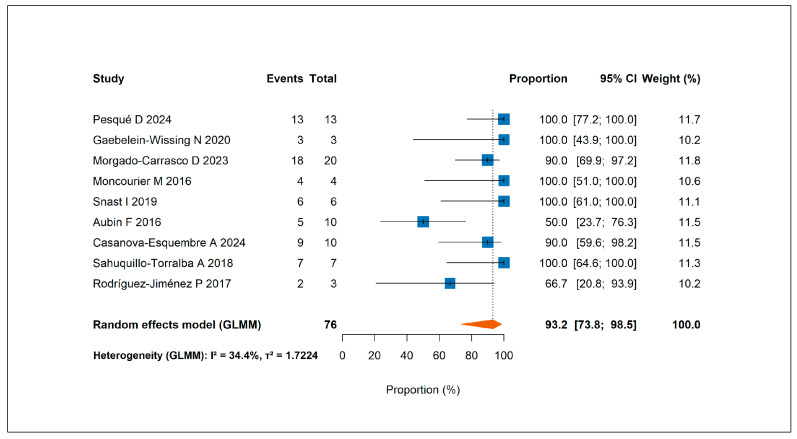
Forest plot showing the pooled rate of at least partial response to omalizumab across all included studies [16,27,30,31,32,35,43,48,49].

An at least partial response was reported in 67 patients, corresponding to a pooled response rate of 93.2% (95% CI, 73.8–98.5%) (Figure 4). A complete response was achieved in 52 patients, with a pooled complete response rate of 68.4% (95% CI, 48.5–83.2%) (Supplementary Materials, Figure S11).

In studies that reported the timing of response, complete improvement was typically observed within 2 to 3 months following initiation of omalizumab. Long-term follow-up durations varied, with reported medians ranging from 6 to 51.4 months. Across these studies, sustained response was generally maintained, with no reports of complete loss of efficacy over time. One study described a decline in response in two patients during treatment, which was managed by increasing the omalizumab dose [30].

Sensitivity analyses by study design revealed no substantial differences in response rates between subgroups (Supplementary Materials, Figure S12a–d).

Adverse events were addressed in six studies. Five studies reported no adverse events [27,30,32,43,49], while one study described a single case of a mild local injection site reaction [35].

**Figure 5:**
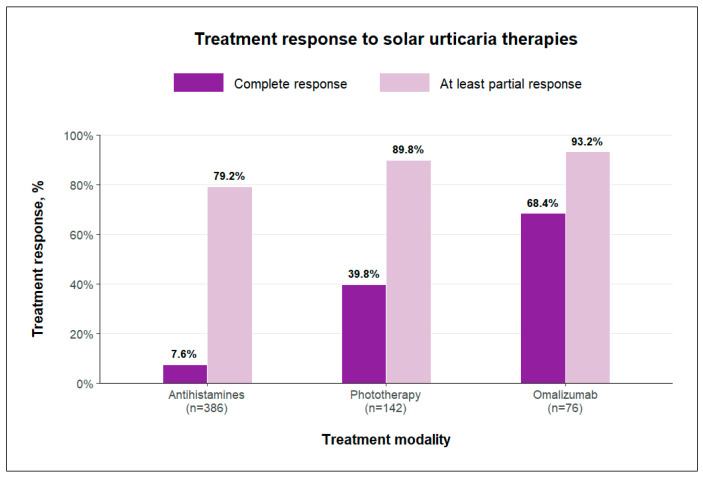
Treatment response rates to solar urticaria therapies by modality, showing proportions of at least partial and complete responses.

A visual summary of at least partial and complete response rates by treatment modality is provided in Figure 5.

#### 3.2.4. Other Treatments

IVIG was evaluated in two studies that met the inclusion criteria, both involving patients with severe and treatment-refractory solar urticaria. A prospective phase II multicenter trial including nine patients reported at least partial response in six (66.7%) at 4 and 12 weeks following a single IVIG infusion. Long-term follow-up was available for only two patients: one maintained clinical response at 48 weeks, while the other relapsed [44]. The second study, a retrospective case series of seven patients, reported a response in five (71%). In this study, the number of IVIG courses ranged from 1 to 3, with intervals of 2–9 months between infusions. Among responders, complete remission was documented for 4 to more than 12 months; however, most patients continued to require additional therapies, such as phototherapy or antihistamines [33]. Across both studies, most patients experienced treatment-related adverse events, generally mild to moderate, with headaches being the most frequently reported symptom.

Cyclosporine was evaluated in a single retrospective case series that met the inclusion criteria, involving 11 patients with severe and treatment-resistant solar urticaria. Clinical improvement was observed in only two patients (18%), and adverse events were reported in five (45%) and led to treatment discontinuation in one case due to chest oppression [34].

Evidence for plasmapheresis was limited to a single case series involving three patients with severe solar urticaria. Complete remission was achieved in one patient, a transient clinical improvement was observed in another, and no response was seen in the third. Adverse events were reported in two patients, including a hypotonic crisis and an anaphylactoid reaction [36].

In addition, an open-label clinical study evaluated the use of oral polypodium leucotomos extract in several patients with various photodermatoses, including four with solar urticaria. Among these four patients, three showed no clinical improvement. No treatment-related adverse events were reported [45].

## 4. Discussion

This systematic review and meta-analysis aimed to evaluate the efficacy of various treatment modalities for solar urticaria, a rare and disabling photodermatosis for which evidence-based therapeutic guidance remains limited. Given the absence of standardized therapeutic algorithms and the reliance on small-scale observational studies, we synthesized available data to estimate pooled response rates and to better characterize the clinical effectiveness of currently reported interventions.

Antihistamines constituted the largest treatment group in our analysis, encompassing 376 patients across 21 studies. This is not surprising, as H_1_ antihistamines have been available for many years and are frequently used as a first-line therapy for solar urticaria. The overall pooled response rate was relatively high, with at least partial improvement reported in 79.2% of patients. However, a complete response was achieved in only 7.6%, underscoring the limited capacity of antihistamines to achieve full disease control in many cases.

A subgroup analysis of patients treated with a combination of antihistamines and LRAs revealed a higher proportion of complete responses. Nevertheless, the number of patients in this subgroup was relatively small (n = 35), and further research is required to validate this observation.

Importantly, even a partial clinical response may be sufficient to restore daily functioning and substantially improve quality of life in many patients. Therefore, given their favorable safety profile, ease of administration, and wide availability, antihistamines remain a rational initial approach in the therapeutic management of patients with solar urticaria.

Phototherapy demonstrated at least a partial response rate of 89.8%, comparable to that of antihistamines. However, a higher proportion of patients (36.6%) achieved complete response, suggesting that phototherapy may offer an advantage in achieving more profound disease control in selected cases. Nevertheless, its practical limitations should be considered. Phototherapy regimens typically require 2 to 3 sessions per week over extended periods, and maintenance therapy is necessary to preserve clinical improvement.

Moreover, although phototherapy was generally well tolerated, more pronounced adverse reactions were reported in this group, including systemic symptoms such as dizziness, throat swelling, and panic-like responses following higher UVA doses.

Importantly, although none of the studies included in this review explicitly commented on the risk of cutaneous malignancies associated with phototherapy, this remains an important consideration. Several earlier reports regarding phototherapy (indicated for dermatological conditions not included or related to solar urticaria) utilized PUVA treatment, which has been associated with an increased risk of non-melanoma skin cancer [50] and, to a lesser extent, melanoma [51], especially with long-term and high-dose exposure. However, the more recent studies in our review employed UVB or UVA1 phototherapy, which has not been conclusively linked to heightened skin cancer risk in the current literature.

Omalizumab was evaluated in 76 patients across 9 studies, representing the smallest treatment group in our analysis. This likely reflects its later introduction into the therapeutic landscape of solar urticaria, as well as its more frequent use in later lines of therapy following failure of other modalities. Despite being typically administered to patients with more severe or treatment-resistant disease, this group exhibited the most impressive treatment outcomes, with partial response achieved in over 93.2% of patients and complete response in 68.4%. These findings suggest that the true efficacy of omalizumab in less refractory cases may be even greater.

Importantly, omalizumab was well tolerated across studies, with only one report of a mild local injection site reaction. However, given that it is a biologic therapy administered by injection and associated with substantial cost, its current use primarily among patients who have not responded to other treatments is understandable.

Several limitations should be acknowledged when interpreting these findings. The most prominent limitation concerns the heterogeneity of the included studies, particularly regarding treatment protocols. This was especially evident in the antihistamine group, where a wide range of agents (e.g., first- and second-generation antihistamines) and dosing regimens (e.g., standard versus updosing) were used. In many studies, the specific type, dose, and duration of antihistamines were not clearly reported, which further complicated comparisons. Similar variability was observed in phototherapy protocols, including differences in the type of phototherapy (e.g., UVA and NB-UVB), treatment frequency, and cumulative doses. In some studies, even within a single cohort, patients received different therapeutic regimens. This lack of consistency significantly limited our ability to perform subgroup analyses based on specific formulations, dosing strategies, or phototherapy protocols. Accordingly, it limits the ability to draw firm conclusions regarding the optimal type and dose of antihistamines, as well as the most effective phototherapy regimen. These limitations further highlight the urgent need for standardized, prospective studies to more reliably evaluate and compare treatment efficacy across modalities.

Another important limitation concerns the strength of the available evidence, which is inherently limited by the study design of the included reports. Most were observational case series of variable methodological quality (Supplementary Materials, Figure S1a–d), with several lacking control groups, randomization, or standardized outcome assessment. While such studies are often the best available evidence for rare conditions like solar urticaria, these methodological limitations limit the strength of the evidence and should be considered when interpreting the findings and comparing treatment effects across studies.

It is also noteworthy that more than half of the included studies were published over a decade ago, including nine that date back more than 30 years. While older case series remain valuable in the context of a rare disease, this temporal distribution further highlights the need for updated prospective studies that reflect current diagnostic criteria, therapeutic standards, and clinical practice patterns.

Additionally, the criteria used to assess treatment response varied substantially across studies. Among those relying on clinical assessment, definitions ranged from reductions in episode frequency or pruritus severity to improvements in quality-of-life scores, and in some cases, the criteria were not clearly specified. In contrast, several studies focused on phototesting outcomes, primarily improvement in MUD. However, MUD improvement does not necessarily correlate with clinical response, and the magnitude of change (e.g., a two- or threefold increase) may not be equally meaningful across patients with low versus high baseline MUD values.

Lastly, we excluded studies with fewer than three patients, which may have led to the omission of relevant treatment experiences and limited the ability to capture the full spectrum of treatment approaches. For example, a case report describing successful treatment of refractory and disabling solar urticaria with the high-affinity anti-IgE antibody ligelizumab—following secondary failure of omalizumab—highlights the potential value of such reports in capturing treatment strategies for rare or complex clinical scenarios [52].

Although this case could not be included in our pooled analysis, it is worth noting that it remains the only reported case worldwide. While the patient achieved clinical benefit with ligelizumab, this agent is currently not available for routine clinical use in solar urticaria. This example also highlights the therapeutic potential of novel and investigational agents and offers hope that future studies will enable the systematic evaluation of such treatments—not only for solar urticaria but also for chronic inducible urticarias.

**Figure 6:**
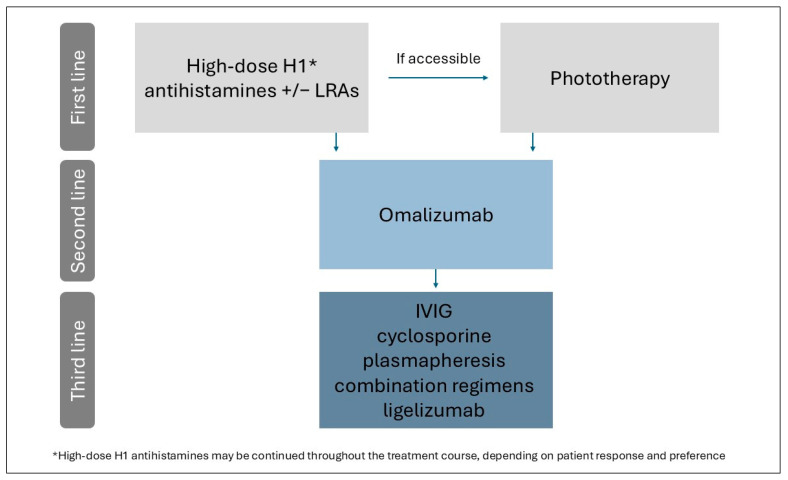
Proposed stepwise treatment algorithm for solar urticaria.

Despite these limitations, our study provides the most comprehensive synthesis to date of treatment outcomes in solar urticaria. The inclusion of a large number of patients and the use of quantitative synthesis enhance the reliability and clinical applicability of our findings, offering pooled estimates of treatment response and safety data. These results may assist in the formulation of a pragmatic, evidence-informed treatment strategy. Accordingly, we propose the following stepwise therapeutic framework based on the aggregated findings of this review (Figure 6).

Given their favorable safety profile, accessibility, and ease of use, high-dose H_1_ antihistamines—either alone or in combination with LRAs—should be considered a first-line treatment.

Phototherapy may be considered as a first- or second-line option when available and feasible, particularly for patients with access to treatment centers and the ability to adhere to frequent treatment sessions.

In cases of inadequate response to these initial treatments, omalizumab represents a highly effective second-line therapy, with consistent response rates across studies and a favorable safety profile.

For patients with refractory disease, additional options may include IVIG, cyclosporine, plasmapheresis, or combination regimens—although current evidence supporting these therapies is limited and primarily based on small case series or individual reports.

This proposed treatment algorithm may assist clinicians in navigating therapeutic choices and lay the groundwork for future efforts to standardize care in solar urticaria.

## 5. Conclusions

This systematic review and meta-analysis provides a comprehensive overview of the current evidence on treatment outcomes in solar urticaria, offering a clearer understanding of the relative efficacy of available therapies. By synthesizing response rates across multiple studies, our findings may assist clinicians both in guiding treatment decisions and in counseling patients more effectively, helping to establish realistic expectations regarding therapeutic outcomes.

## Figures and Tables

**Table 1:** Main characteristics of the studies included in the systematic review.

Author, Year	Country	Study Design	No of Patients Included	Type of Treatment
Du-Thanh et al., 2013 [4]	France	Case series	29	Antihistamines
Gaebelein-Wissing et al., 2020 [16]	Germany	Case series	23	Antihistamines Phototherapy Omalizumab
Imamura et al., 2024 [17]	Japan	Case series	26	Antihistamines Phototherapy
Parrish et al., 1982 [37]	USA	Open-label interventional study	8	Antihistamines Phototherapy
Monfrecola et al., 2000 [18]	Italy	Case series	57	Antihistamines Phototherapy
Pesqué et al., 2024 [49]	Spain	Cohort study	41	Antihistamines Omalizumab
Uetsu et al., 2020 [19]	Japan	Case series	11	Antihistamines
Fityan et al., 2018 [20]	UK	Case series	5	Antihistamines
Silpa-Archa et al., 2016 [21]	Thailand	Case series	13	Antihistamines
Chong et al., 2004 [22]	Singapore	Case series	19	Antihistamines
Monfrecola et al., 1990 [23]	Italy	Case series	3	Antihistamines
Grundmann et al., 2008 [24]	Germany	Case series	4	Antihistamines + LRA
Faurschou et al., 2008 [38]	Denmark	Open-label interventional study	3	Antihistamines
Beattie et al., 2003 [25]	UK	Case series	34	Antihistamines
Monfrecola et al., 2000 [39]	Italy	Open-label interventional study	7	Antihistamines
Michell et al., 1980 [46]	UK	Randomized clinical trial	8	Antihistamines
Reinauer et al., 1993 [26]	Germany	Case series	3	Antihistamines
Casanova-Esquembre et al., 2024 [48]	Spain	Case-control study	59	Antihistamines Omalizumab
Bilsland et al., 1991 [47]	UK	Randomized clinical trial	6	Antihistamines
Snast et al., 2019 [27]	Israel	Case series	23	Antihistamines + LRA Omalizumab
Levi et al., 2015 [5]	Israel	Case series	8	Antihistamines + LRA
Lyons et al., 2019 [28]	USA	Case series	3	Phototherapy
Calzavara-Pinton et al., 2012 [40]	Italy	Open-label interventional study	39	Phototherapy
Bernhard et al., 1984 [41]	USA	Open-label interventional study	5	Phototherapy
Beissert et al., 2000 [6]	Germany	Case series	3	Phototherapy
Keahey et al., 1984 [42]	USA	Open-label interventional study	3	Phototherapy
Addo et al., 1987 [7]	UK	Open-label interventional study	4	Phototherapy
Chicharro et al., 2018 [29]	Spain	Case series	8	Phototherapy
Morgado-Carrasco et al., 2023 [30]	Spain	Case series	20	Omalizumab
Moncourier et al., 2016 [31]	France	Case series	4	Omalizumab
Aubin et al., 2016 [43]	France	Open-label interventional study	10	Omalizumab
Sahuquillo-Torralba et al., 2018 [32]	Spain	Case series	7	Omalizumab
Rodríguez-Jiménez et al., 2017 [35]	Spain	Case series	3	Omalizumab
Adamski et al., 2011 [33]	France	Case series	7	IVIG
Aubin et al., 2014 [44]	France	Open-label interventional study	9	IVIG
Hurabielle et al., 2015 [34]	France	Case series	11	Cyclosporine
Leenutaphong et al., 1991 [36]	Germany	Case series	3	Plasmapheresis
Caccialanza et al., 2011 [45]	Italy	Open-label interventional study	4	Polypodium leucotomos

Abbreviations: LRA, leukotriene receptor antagonist; IVIG, intravenous immunoglobulin.

**Table 2:** Summary of treatment response assessment methods across included studies.

	Clinical Assessment	Phototesting	Both Clinical Assessment and Phototesting	Not Specified
No of studies	14	6	15	3
No of patients	251	30	165	87

## Data Availability

The data presented in this study are available on request from the corresponding author.

## Supplementary Materials

The following supporting information can be downloaded at https://www.mdpi.com/article/10.3390/jcm14165736/s1, Table S1: Detailed overview of treatment response assessment methods across included studies; Figure S1a: Methodological quality assessment of included case series studies (JBI critical appraisal tool); Figure S1b: Methodological quality assessment of included open-label interventional studies (JBI critical appraisal tool); Figure S1c: Methodological quality assessment of included randomized controlled trials and cohort study (JBI critical appraisal tools); Figure S1d: Methodological quality assessment of the included case–control study (JBI critical appraisal tool); Figure S2: Forest plot showing the pooled rate of complete response to antihistamines across all included studies; Figure S3a: Forest plot showing the pooled rate of at least partial response to antihistamines alone (without LRAs); Figure S3b: Forest plot showing the pooled rate of complete response to antihistamines alone (without LRAs); Figure S4a: Forest plot showing the pooled rate of at least partial response to antihistamines and LRAs; Figure S4b: Forest plot showing the pooled rate of complete response to antihistamines and LRAs; Figure S5a: Forest plot showing the pooled rate of at least partial response to antihistamines across case series included; Figure S5b: Forest plot showing the pooled rate of complete response to antihistamines across case series included; Figure S5c: Forest plot showing the pooled rate of at least partial response to antihistamines across all studies, excluding case series; Figure S5d: Forest plot showing the pooled rate of complete response to antihistamines across all studies, excluding case series; Figure S6: Forest plot showing the pooled rate of complete response to phototherapy across all included studies; Figure S7: Forest plot showing the pooled rate of at least partial response to UVA rush hardening; Figure S8: Forest plot showing the pooled rate of at least partial response to UVA without rush hardening; Figure S9: Forest plot showing the pooled rate of at least partial response to UVB; Figure S10a: Forest plot showing the pooled rate of at least partial response to phototherapy across case series included; Figure S10b: Forest plot showing the pooled rate of complete response to phototherapy across case series included; Figure S10c: Forest plot showing the pooled rate of at least partial response to phototherapy across all studies, excluding case series; Figure S10d: Forest plot showing the pooled rate of complete response to phototherapy across all studies, excluding case series; Figure S11: Forest plot showing the pooled rate of complete response to omalizumab across all included studies; Figure S12a: Forest plot showing the pooled rate of at least partial response to omalizumab across case series included; Figure S12b: Forest plot showing the pooled rate of complete response to omalizumab across case series included; Figure S12c: Forest plot showing the pooled rate of at least partial response to omalizumab across all studies, excluding case series; Figure S12d: Forest plot showing the pooled rate of complete response to omalizumab across all studies, excluding case series.

## Abbreviations

The following abbreviations are used in this manuscript:

UVA	Ultraviolet A
VL	Visible light
LRAs	Leukotriene receptor antagonists
NB-UVB	Narrowband UVB
IVIG	Intravenous immunoglobulin
PRISMA	Preferred Reporting Items for Systematic Reviews and Meta-Analyses
RCTs	Randomized controlled trials
JBI	Joanna Briggs Institute
