# Supplementary material for: Efficacy of Therapies for Solar Urticaria: A Systematic Review and Meta-Analysis
Source: J Clin Med. 2025 Aug 13;14(16):5736. doi: 10.3390/jcm14165736 (PMC12386910; doi:10.3390/jcm14165736)
Supplement: Supplementary file 1 [file jcm-14-05736-s001.zip › Table S1.pdf]

Table S1: Detailed overview of treatment response assessment methods across included studies

Author, Year	Treatment response assessment methods	Additional Notes
Du-Thanh et al. 2013 [4]	Clinical assessment	"Dermatologist's observations"; no further details provided
Gaebelein-Wissing et al. 2020 [16]	Not specified	"Good results", "insufficient", "remission"; no further details provided
Imamura et al. 2024 [17]	Clinical assessment or phototesting	Complete response was defined as absence of urticaria in daily life or during photo-provocation. Partial response included cases where MUD was prolonged but urticaria still appeared upon photo-provocation
Parrish et al. 1982 [37]	Both clinical assessment and phototesting	"Patients' reports of their tolerance to sunlight exposure during the summer months", no further details provided
Monfrecola et al. 2000 [18]	Both clinical assessment and phototesting	"Reduce the intensity of wheals and erythema ", "improvement of symptoms"<br>MUD response was categorized as follows: MUDs not detectable at doses >20-fold the baseline, MUD increases of 10- to 20-fold, and MUD increases of 4- to 8-fold
Pesqué et al. 2024 [49]	Clinical assessment	Urticaria Control Test (UCT): partial response (UCT 12–15), complete response (UCT = 16)
Uetsu et al. 2020 [19]	Both clinical assessment and phototesting	"Effective"- no urticaria after sun exposure,"control the disease", "refractory", no further details provided
Fityan et al. 2018 [20]	Not specified	"Significant improvement", "near complete resolution", " failed to respond", no further details provided
Silpa-Archa et al. 2016 [21]	Clinical assessment	Complete response was defined as no cutaneous symptoms after sun exposure
Chong et al. 2004 [22]	Clinical assessment	"Partial improvement in symptoms" – "reduce the intensity of wheals"
Monfrecola et al. 1990 [23]	Phototesting	Partial response - twofold increase of MUD<br>Complete response - marked increase of the minimal time required to produce wheal
Grundmann et al. 2008 [24]	Both clinical assessment and phototesting	"Free of urticarial skin lesions", " feeble itch ", "photoprovocation without any urticarial eruptions", "photoprovocation with only mild pruritus", no further details provided
Faurschou et al. 2008 [38]	Phototesting	Increase in MUD values, no further details provided
Beattie et al. 2003 [25]	Clinical assessment	"Very good benefit", "some symptom control", "benefit was doubtful", no further details provided
Monfrecola et al. 2000 [39]	Phototesting	Increase in MUD values, no further details provided
Michell et al. 1980 [46]	Phototesting	"Significant reduction in weal size"
Reinauer et al. 1993 [26]	Phototesting	Increase in MUD values, presence of erythema after photoprovocation
Casanova-Esquembre et al. 2024 [48]	Not specified	"Failed treatment", "complete response", no further details provided
Bilsland et al. 1991 [47]	Phototesting	Increase in MUD values, no further details provided
Snast et al. 2019 [27]	Clinical assessment	"Treatment failure was defined as a treatment that did not enable patients to remain free or almost free of symptoms outdoors"
Levi et al. 2015 [5]	Both clinical assessment and phototesting	"Complete remission of symptoms and could return to normal outdoor activity in daylight", "only subjective response", Increase in MUD values

Lyons et al. 2019 [28]	Clinical assessment	"Improvement of symptoms", "good control of symptoms", no further details provided
Calzavara-Pinton et al. 2012 [40]	Clinical assessment	" SU after sun exposure", "episode of mild erythema", "episode of painful marked erythema", no further details provided
Bernhard et al. 1984 [41]	Both clinical assessment and phototesting	Increase in MUD values , "increased tolerance to Sunlight"
Beissert et al. 2000 [6]	Both clinical assessment and phototesting	"No wheals developed, slight pruritus in sun-exposed areas", "symptom free", "no urticarial reactions were provoked"
Keahey et al. 1984 [42]	Clinical assessment	The ability to stay in direct noon sunlight for at least 2 h
Addo et al. 1987 [7]	Clinical assessment	Satisfactory—no reaction on exposure to sunlight; moderately satisfactory—minimal reaction occurring on less than three occasions following exposure to sunlight; unsatisfactory—frequent reactions (on more than three occasions) requiring topical steroids and sunscreens
Chicharro et al. 2018 [29]	Clinical assessment	Skindex-29 - a questionnaireon health-related quality of life and another nonvalidated questionnaire on treatment effectiveness and patient satisfaction
Morgado-Carrasco et al. 2023 [30]	Clinical assessment	Clinical response was evaluated by using the Urticaria Activity Score (UAS7) and UCT. Complete cresponse was defined as having a UAS7 equal to zero
Moncourier et al. 2016 [31]	Both clinical assessment and phototesting	" clinical improvement"- no further details provided, increase in MUD values
Aubin et al. 2016 [43]	Both clinical assessment and phototesting	Dermatology Life Quality Index score (50% improvement from baseline), wheal-free: (urticarial activity score over 7 days=0), increase in MUD values
Sahuquillo-Torralba et al. 2018 [32]	Both clinical assessment and phototesting	Complete response was considered when patients were asymptomatic during their daily activities and prolonged solar exposures, together with a negative Phototest. Partial response was considered when patients were asymptomatic duringtheir daily activities with improvement or negative Phototest, but with clinical manifestations to prolonged solar exposures
Rodríguez-Jiménez et al. 2017 [35]	Both clinical assessment and phototesting	Skindex-29 in one patient, no further details provided
Adamski et al. 2011 [33]	Both clinical assessment and phototesting	Increase in MUD values, clinical complete remission of SU - no further details provided
Aubin et al. 2014 [44]	Both clinical assessment and phototesting	MUD, DLQI, disease activity (appearance or not of at least 1 SU flare during the 7 days before the in-clinic evaluation), and intensity of SU measured by a visual analog scale score ranging from 0 (no SU) to 10 (maximal conceivable intensity of SU)
Hurabielle et al. 2015 [34]	Clinical assessment	"No modification of SU symptoms", partial response
Leenutaphong et al. 1991 [36]	Both clinical assessment and phototesting	Increase in MUD values, tolerance of intense sunshine
Caccialanza et al. 2011 [45]	Clinical assessment	Benefitial during sunlight exposure
