# Supplementary material for: Efficacy of Therapies for Solar Urticaria: A Systematic Review and Meta-Analysis
Source: J Clin Med. 2025 Aug 13;14(16):5736. doi: 10.3390/jcm14165736 (PMC12386910; doi:10.3390/jcm14165736)
Supplement: Supplementary file 1 [file jcm-14-05736-s001.zip › figS1a case series.pptx]

## Slide 1
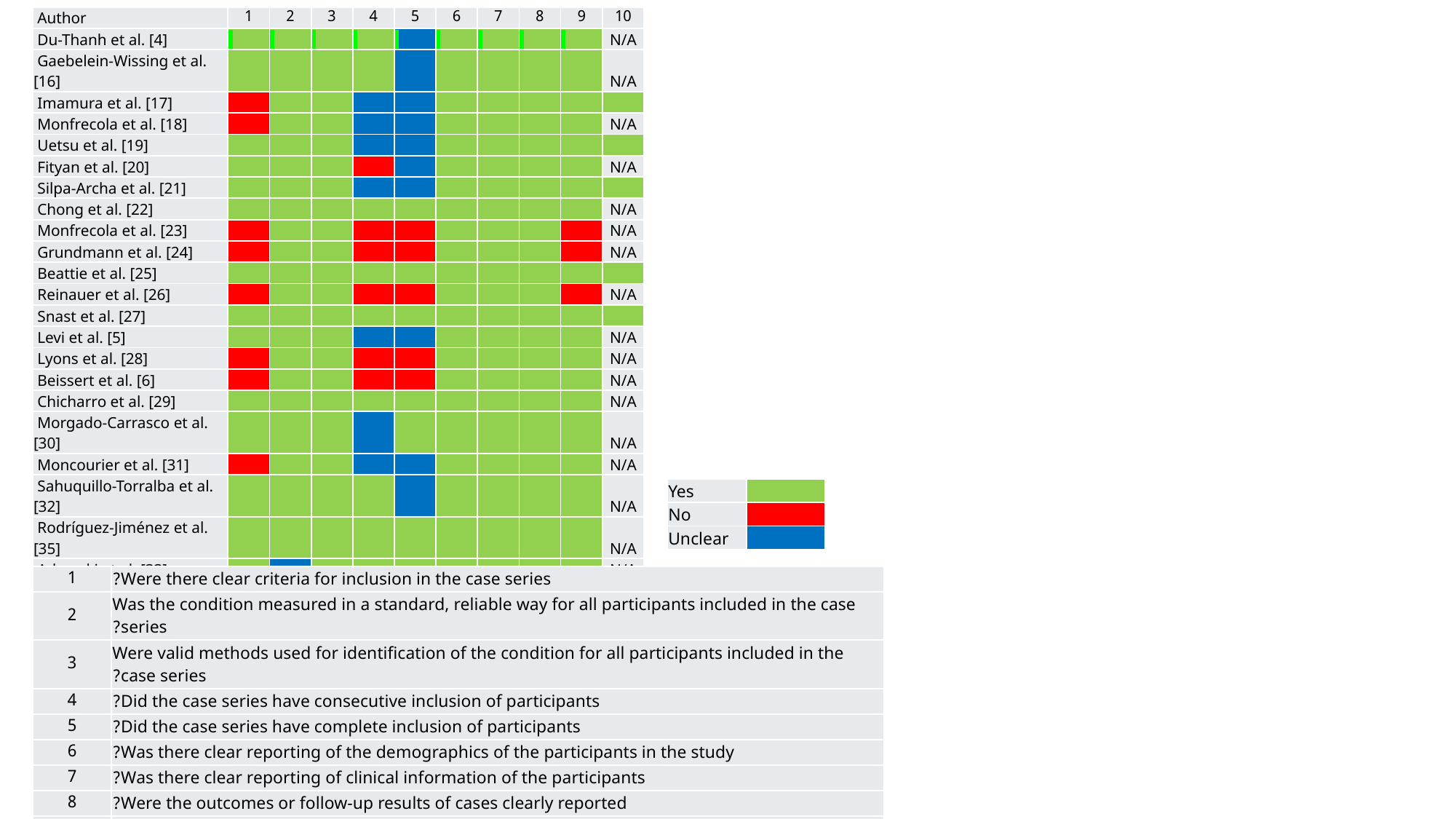

| Author | 1 | 2 | 3 | 4 | 5 | 6 | 7 | 8 | 9 | 10 |
| --- | --- | --- | --- | --- | --- | --- | --- | --- | --- | --- |
| Du-Thanh et al. [4] | | | | | | | | | | N/A |
| Gaebelein-Wissing et al. [16] | | | | | | | | | | N/A |
| Imamura et al. [17] | | | | | | | | | | |
| Monfrecola et al. [18] | | | | | | | | | | N/A |
| Uetsu et al. [19] | | | | | | | | | | |
| Fityan et al. [20] | | | | | | | | | | N/A |
| Silpa-Archa et al. [21] | | | | | | | | | | |
| Chong et al. [22] | | | | | | | | | | N/A |
| Monfrecola et al. [23] | | | | | | | | | | N/A |
| Grundmann et al. [24] | | | | | | | | | | N/A |
| Beattie et al. [25] | | | | | | | | | | |
| Reinauer et al. [26] | | | | | | | | | | N/A |
| Snast et al. [27] | | | | | | | | | | |
| Levi et al. [5] | | | | | | | | | | N/A |
| Lyons et al. [28] | | | | | | | | | | N/A |
| Beissert et al. [6] | | | | | | | | | | N/A |
| Chicharro et al. [29] | | | | | | | | | | N/A |
| Morgado-Carrasco et al. [30] | | | | | | | | | | N/A |
| Moncourier et al. [31] | | | | | | | | | | N/A |
| Sahuquillo-Torralba et al. [32] | | | | | | | | | | N/A |
| Rodríguez-Jiménez et al. [35] | | | | | | | | | | N/A |
| Adamski et al. [33] | | | | | | | | | | N/A |
| Hurabielle et al. [34] | | | | | | | | | | N/A |
| Leenutaphong et al. [36] | | | | | | | | | | N/A |
| Yes | |
| --- | --- |
| No | |
| Unclear | |
| 1 | Were there clear criteria for inclusion in the case series? |
| --- | --- |
| 2 | Was the condition measured in a standard, reliable way for all participants included in the case series? |
| 3 | Were valid methods used for identification of the condition for all participants included in the case series? |
| 4 | Did the case series have consecutive inclusion of participants? |
| 5 | Did the case series have complete inclusion of participants? |
| 6 | Was there clear reporting of the demographics of the participants in the study? |
| 7 | Was there clear reporting of clinical information of the participants? |
| 8 | Were the outcomes or follow-up results of cases clearly reported? |
| 9 | Was there clear reporting of the presenting sites'/clinics' demographic information? |
| 10 | Was statistical analysis appropriate? |
