# Supplementary material for: Efficacy of Therapies for Solar Urticaria: A Systematic Review and Meta-Analysis
Source: J Clin Med. 2025 Aug 13;14(16):5736. doi: 10.3390/jcm14165736 (PMC12386910; doi:10.3390/jcm14165736)
Supplement: Supplementary file 1 [file jcm-14-05736-s001.zip › figS1b OL.pptx]

## Slide 1
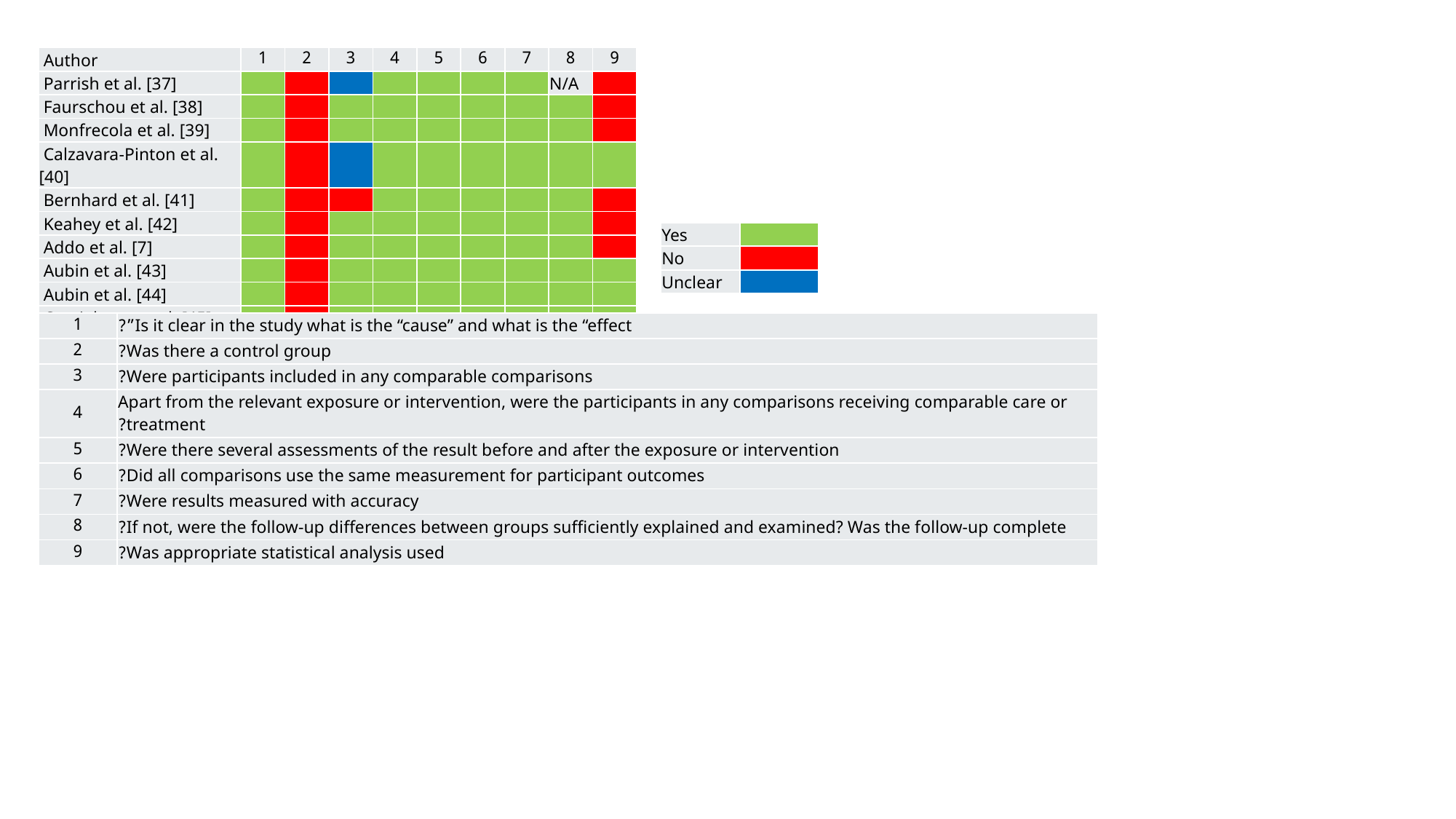

| Author | 1 | 2 | 3 | 4 | 5 | 6 | 7 | 8 | 9 |
| --- | --- | --- | --- | --- | --- | --- | --- | --- | --- |
| Parrish et al. [37] | | | | | | | | N/A | |
| Faurschou et al. [38] | | | | | | | | | |
| Monfrecola et al. [39] | | | | | | | | | |
| Calzavara-Pinton et al. [40] | | | | | | | | | |
| Bernhard et al. [41] | | | | | | | | | |
| Keahey et al. [42] | | | | | | | | | |
| Addo et al. [7] | | | | | | | | | |
| Aubin et al. [43] | | | | | | | | | |
| Aubin et al. [44] | | | | | | | | | |
| Caccialanza et al. [45] | | | | | | | | | |
| Yes | |
| --- | --- |
| No | |
| Unclear | |
| 1 | Is it clear in the study what is the “cause” and what is the “effect”? |
| --- | --- |
| 2 | Was there a control group? |
| 3 | Were participants included in any comparable comparisons? |
| 4 | Apart from the relevant exposure or intervention, were the participants in any comparisons receiving comparable care or treatment? |
| 5 | Were there several assessments of the result before and after the exposure or intervention? |
| 6 | Did all comparisons use the same measurement for participant outcomes? |
| 7 | Were results measured with accuracy? |
| 8 | If not, were the follow-up differences between groups sufficiently explained and examined? Was the follow-up complete? |
| 9 | Was appropriate statistical analysis used? |
