# Supplementary material for: Efficacy of Therapies for Solar Urticaria: A Systematic Review and Meta-Analysis
Source: J Clin Med. 2025 Aug 13;14(16):5736. doi: 10.3390/jcm14165736 (PMC12386910; doi:10.3390/jcm14165736)
Supplement: Supplementary file 1 [file jcm-14-05736-s001.zip › figS1c RCT and cohort.pptx]

## Slide 1
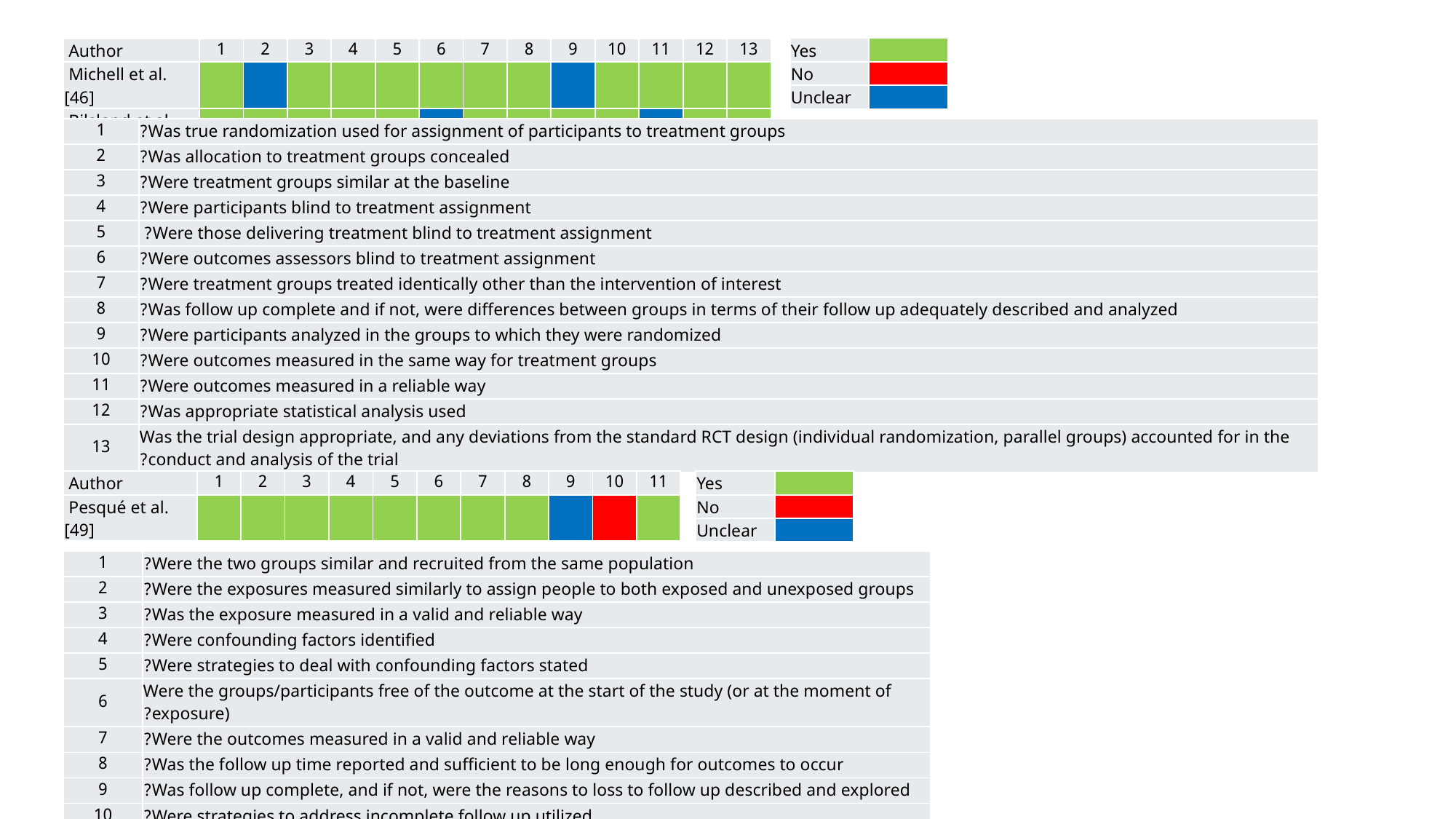

| Yes | |
| --- | --- |
| No | |
| Unclear | |
| Author | 1 | 2 | 3 | 4 | 5 | 6 | 7 | 8 | 9 | 10 | 11 | 12 | 13 |
| --- | --- | --- | --- | --- | --- | --- | --- | --- | --- | --- | --- | --- | --- |
| Michell et al. [46] | | | | | | | | | | | | | |
| Bilsland et al. [48] | | | | | | | | | | | | | |
| 1 | Was true randomization used for assignment of participants to treatment groups? |
| --- | --- |
| 2 | Was allocation to treatment groups concealed? |
| 3 | Were treatment groups similar at the baseline? |
| 4 | Were participants blind to treatment assignment? |
| 5 | Were those delivering treatment blind to treatment assignment? |
| 6 | Were outcomes assessors blind to treatment assignment? |
| 7 | Were treatment groups treated identically other than the intervention of interest? |
| 8 | Was follow up complete and if not, were differences between groups in terms of their follow up adequately described and analyzed? |
| 9 | Were participants analyzed in the groups to which they were randomized? |
| 10 | Were outcomes measured in the same way for treatment groups? |
| 11 | Were outcomes measured in a reliable way? |
| 12 | Was appropriate statistical analysis used? |
| 13 | Was the trial design appropriate, and any deviations from the standard RCT design (individual randomization, parallel groups) accounted for in the conduct and analysis of the trial? |
| Author | 1 | 2 | 3 | 4 | 5 | 6 | 7 | 8 | 9 | 10 | 11 |
| --- | --- | --- | --- | --- | --- | --- | --- | --- | --- | --- | --- |
| Pesqué et al. [49] | | | | | | | | | | | |
| Yes | |
| --- | --- |
| No | |
| Unclear | |
| 1 | Were the two groups similar and recruited from the same population? |
| --- | --- |
| 2 | Were the exposures measured similarly to assign people to both exposed and unexposed groups? |
| 3 | Was the exposure measured in a valid and reliable way? |
| 4 | Were confounding factors identified? |
| 5 | Were strategies to deal with confounding factors stated? |
| 6 | Were the groups/participants free of the outcome at the start of the study (or at the moment of exposure)? |
| 7 | Were the outcomes measured in a valid and reliable way? |
| 8 | Was the follow up time reported and sufficient to be long enough for outcomes to occur? |
| 9 | Was follow up complete, and if not, were the reasons to loss to follow up described and explored? |
| 10 | Were strategies to address incomplete follow up utilized? |
| 11 | Was appropriate statistical analysis used? |
