# Supplementary material for: Efficacy of Therapies for Solar Urticaria: A Systematic Review and Meta-Analysis
Source: J Clin Med. 2025 Aug 13;14(16):5736. doi: 10.3390/jcm14165736 (PMC12386910; doi:10.3390/jcm14165736)
Supplement: Supplementary file 1 [file jcm-14-05736-s001.zip › figS1d case control.pptx]

## Slide 1
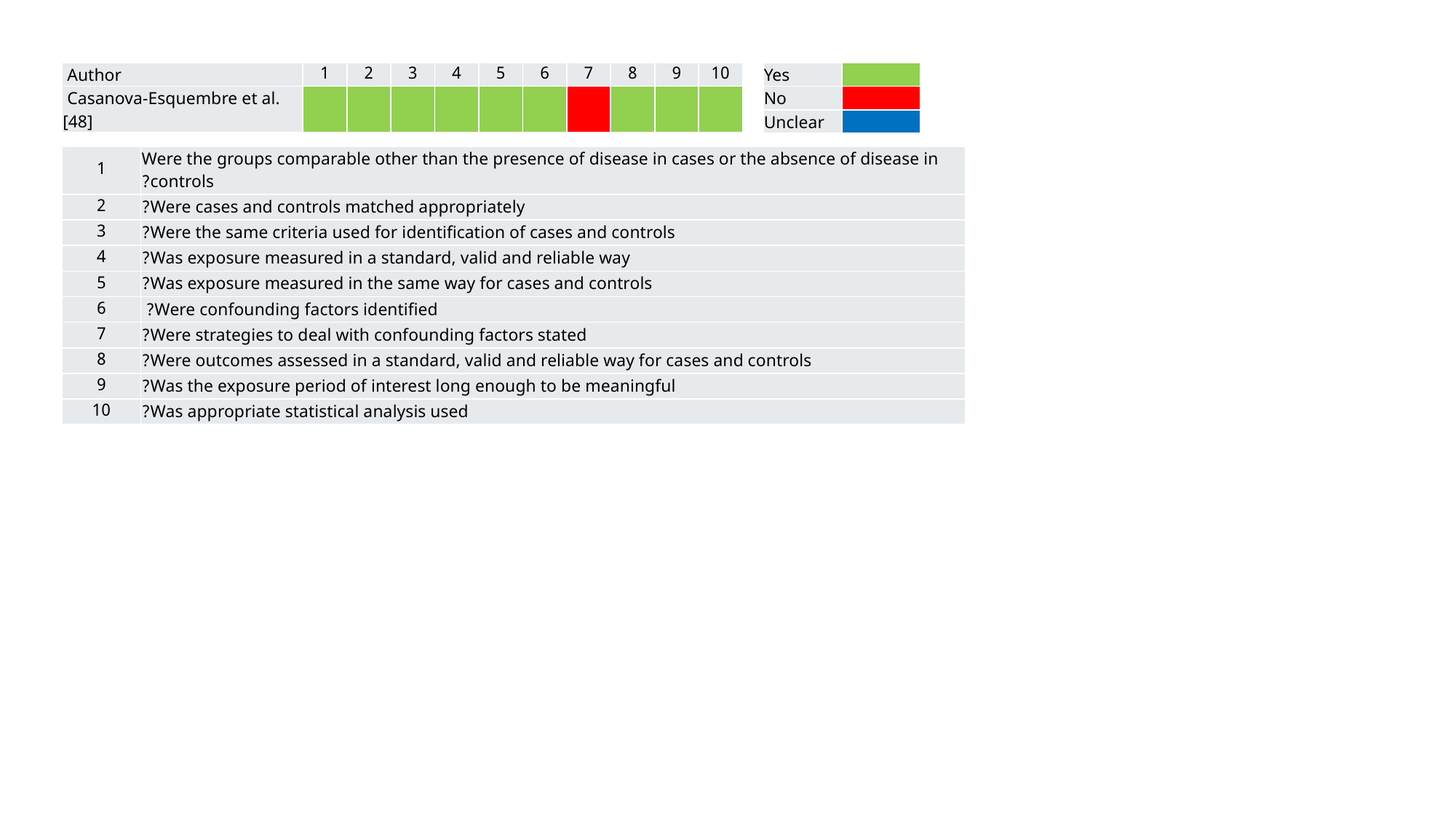

| Author | 1 | 2 | 3 | 4 | 5 | 6 | 7 | 8 | 9 | 10 |
| --- | --- | --- | --- | --- | --- | --- | --- | --- | --- | --- |
| Casanova-Esquembre et al. [48] | | | | | | | | | | |
| Yes | |
| --- | --- |
| No | |
| Unclear | |
| 1 | Were the groups comparable other than the presence of disease in cases or the absence of disease in controls? |
| --- | --- |
| 2 | Were cases and controls matched appropriately? |
| 3 | Were the same criteria used for identification of cases and controls? |
| 4 | Was exposure measured in a standard, valid and reliable way? |
| 5 | Was exposure measured in the same way for cases and controls? |
| 6 | Were confounding factors identified? |
| 7 | Were strategies to deal with confounding factors stated? |
| 8 | Were outcomes assessed in a standard, valid and reliable way for cases and controls? |
| 9 | Was the exposure period of interest long enough to be meaningful? |
| 10 | Was appropriate statistical analysis used? |
