# Supplementary figures and images for: Efficacy of Therapies for Solar Urticaria: A Systematic Review and Meta-Analysis
Source: J Clin Med. 2025 Aug 13;14(16):5736. doi: 10.3390/jcm14165736 (PMC12386910; doi:10.3390/jcm14165736)

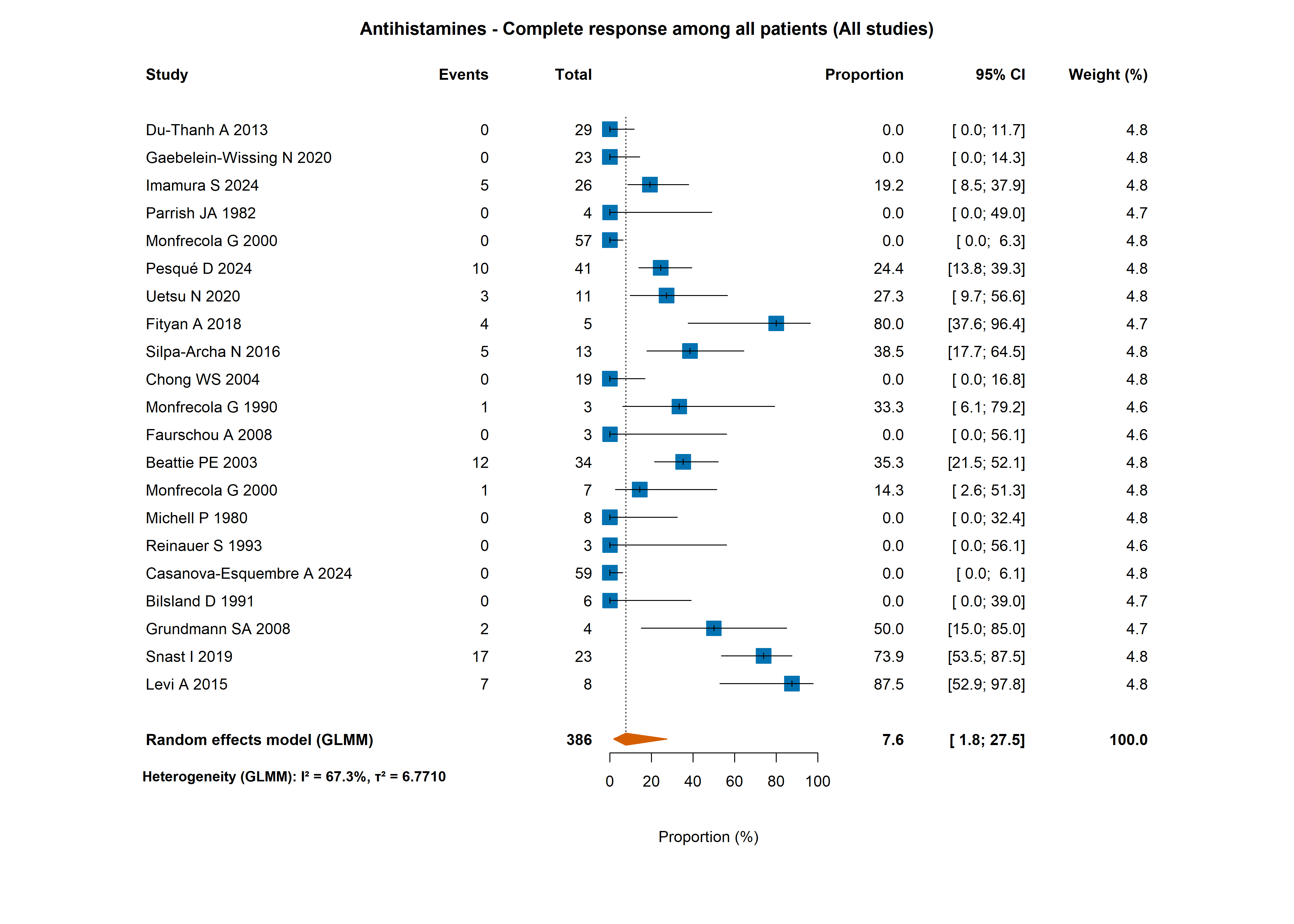

Supplement: Supplementary file 1 [file jcm-14-05736-s001.zip › figS2.png]

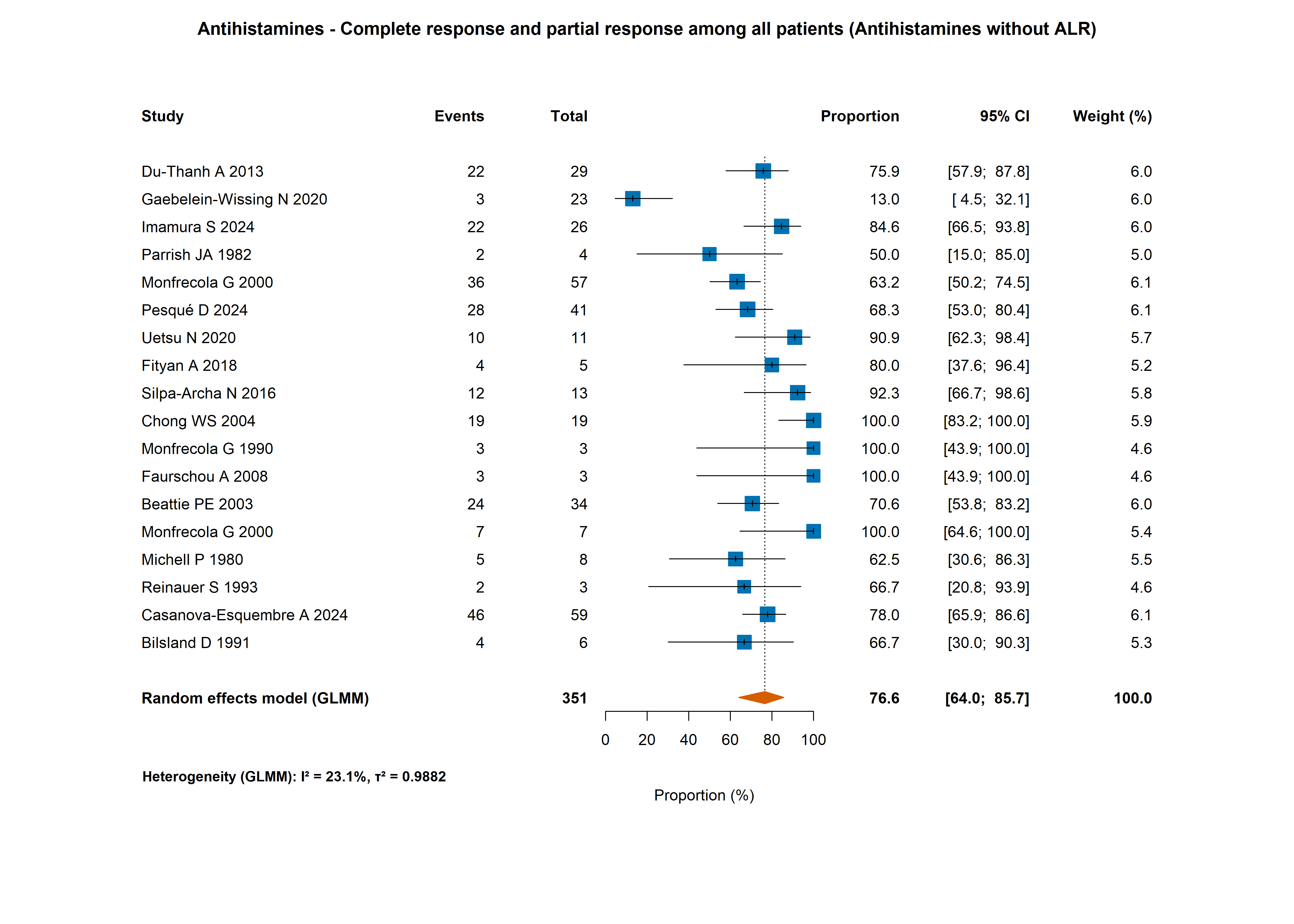

Supplement: Supplementary file 1 [file jcm-14-05736-s001.zip › figS3a.png]

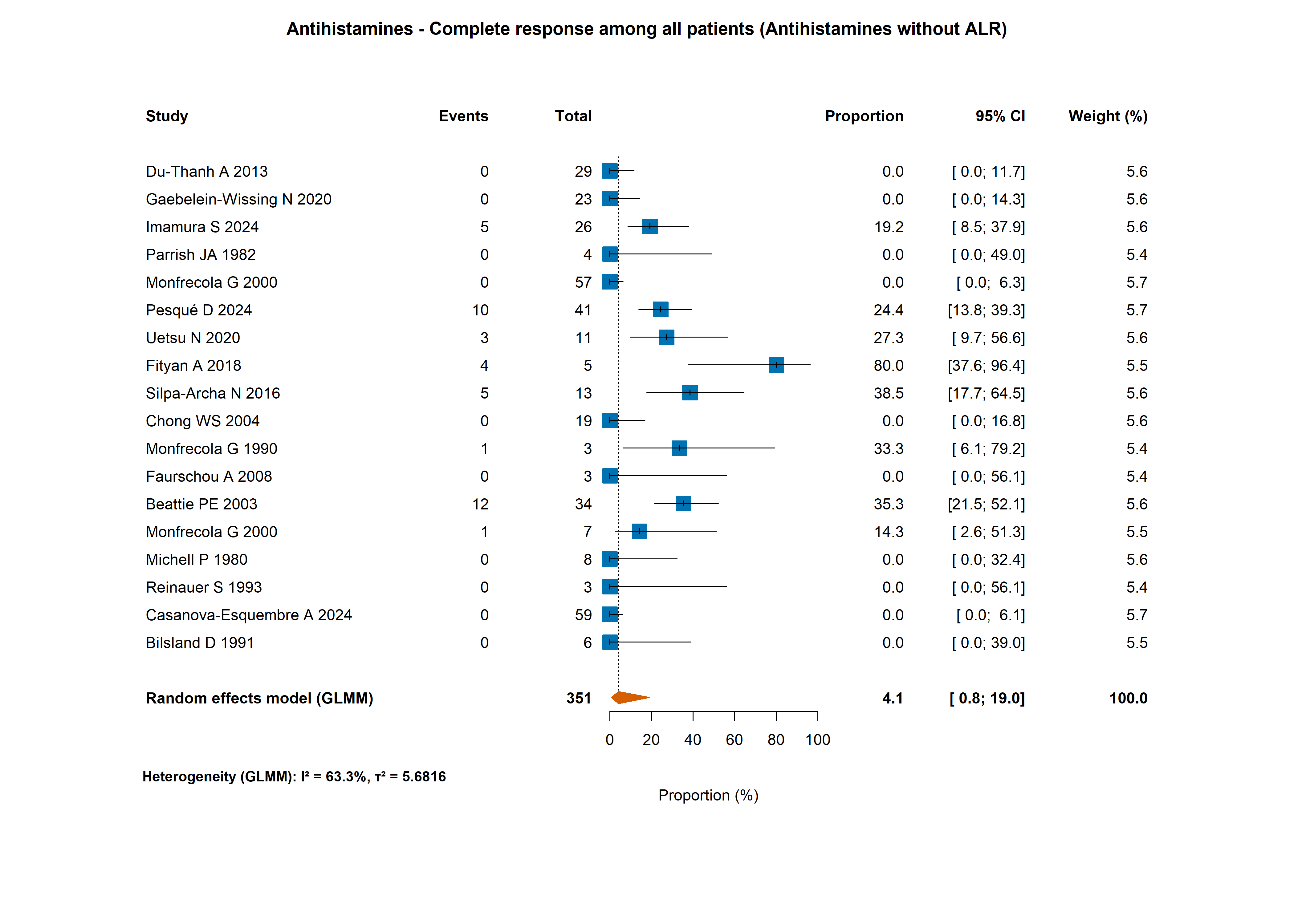

Supplement: Supplementary file 1 [file jcm-14-05736-s001.zip › figS3b.png]

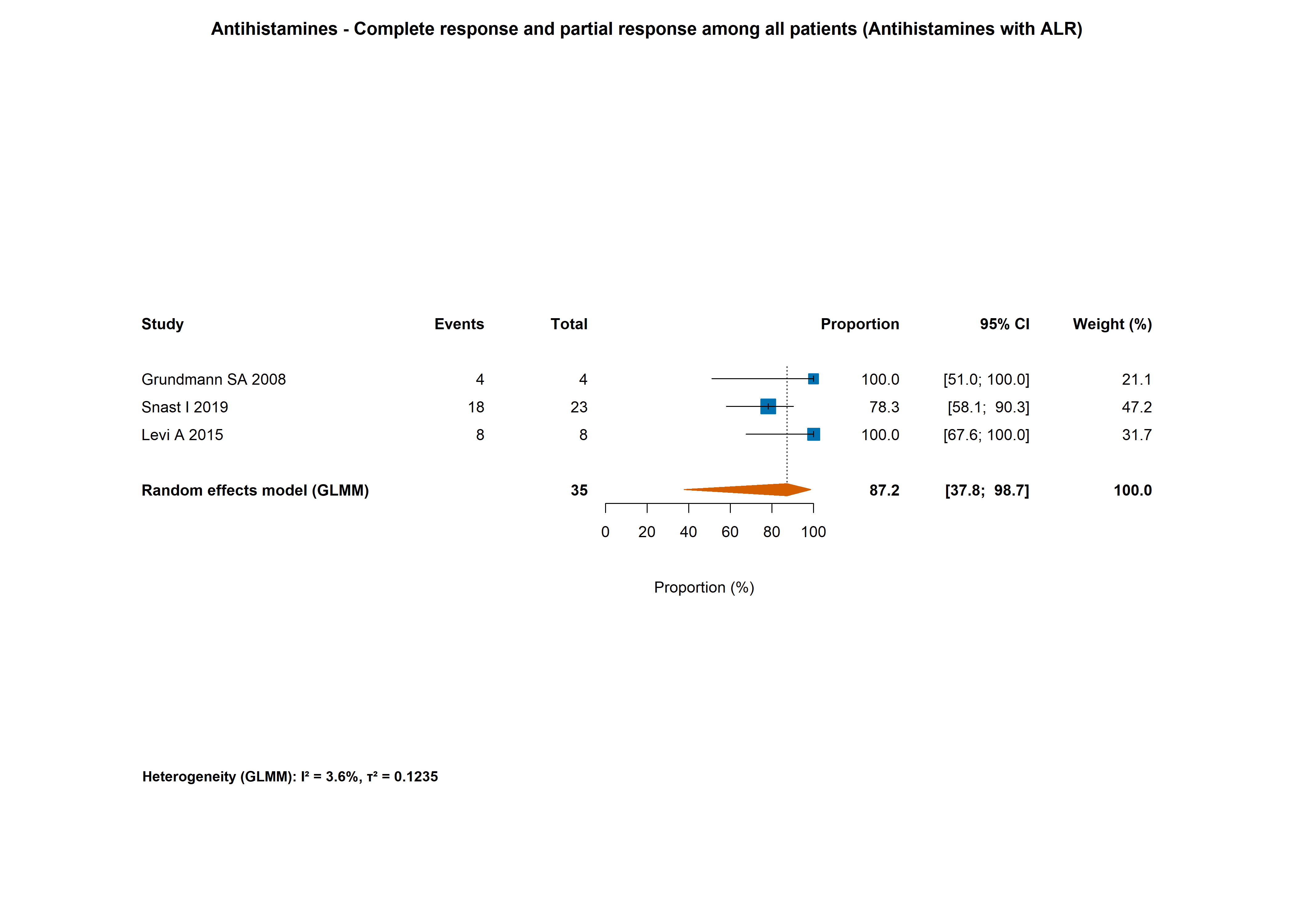

Supplement: Supplementary file 1 [file jcm-14-05736-s001.zip › figS4a.png]

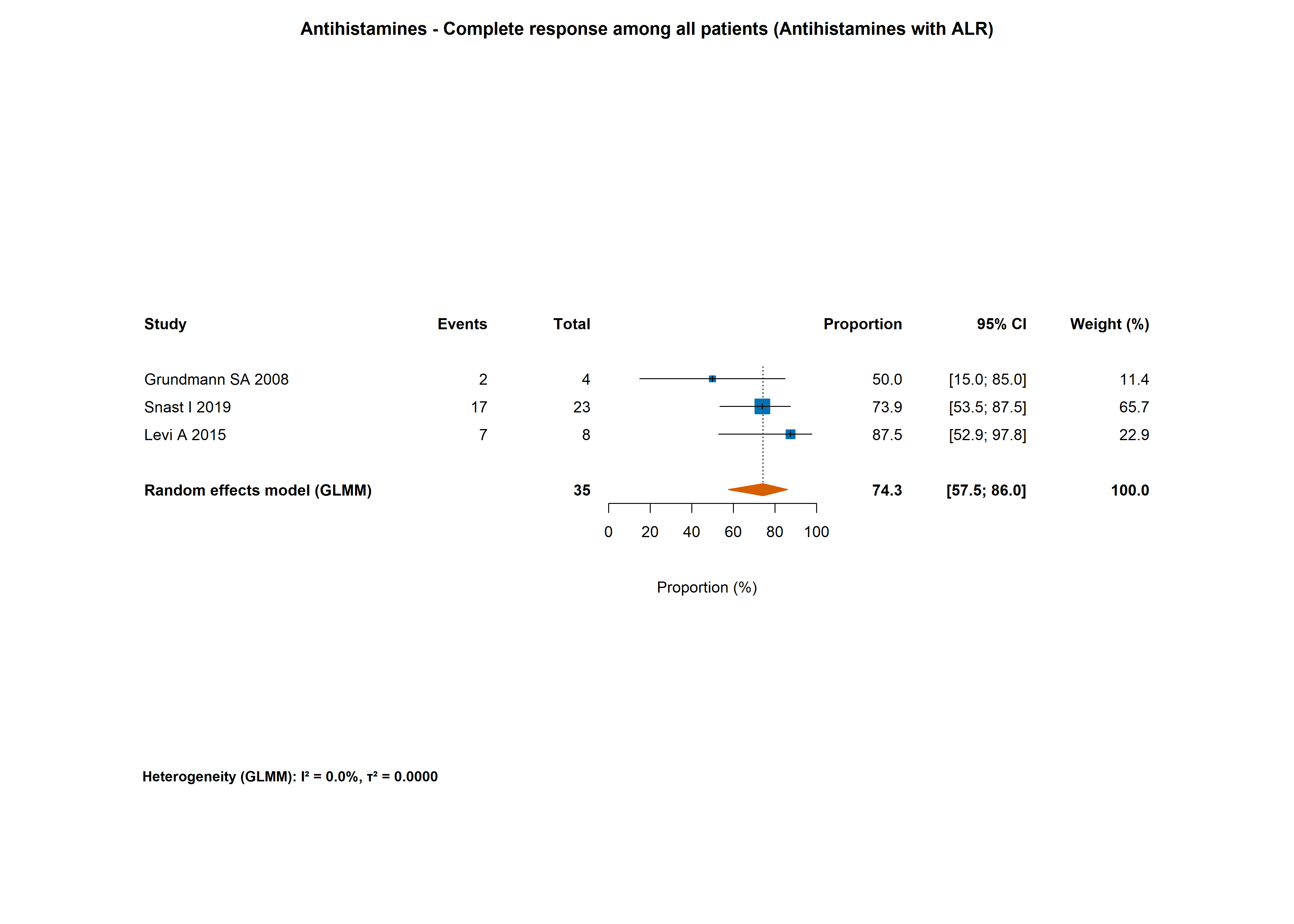

Supplement: Supplementary file 1 [file jcm-14-05736-s001.zip › figS4b.png]

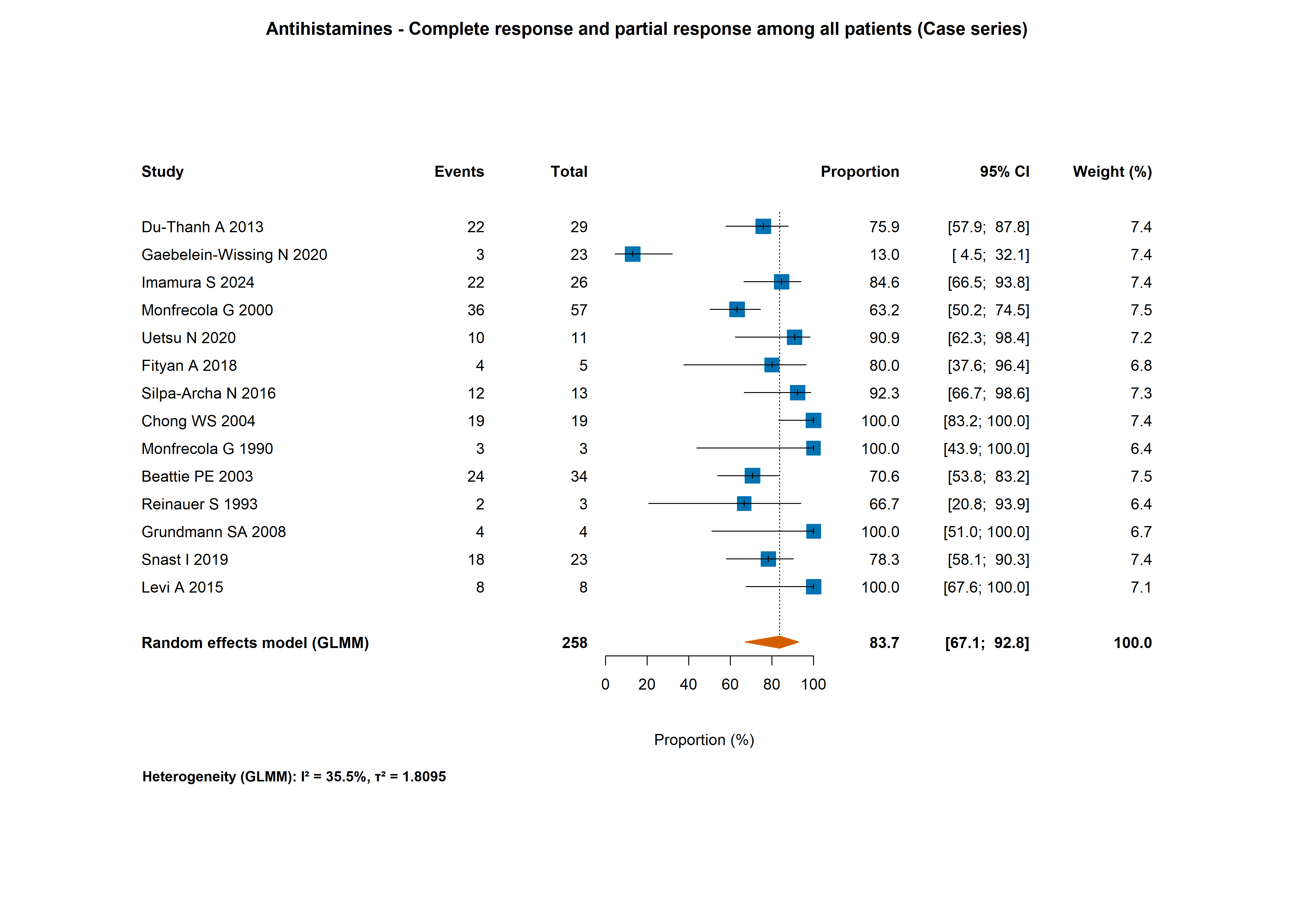

Supplement: Supplementary file 1 [file jcm-14-05736-s001.zip › figS5a.png]

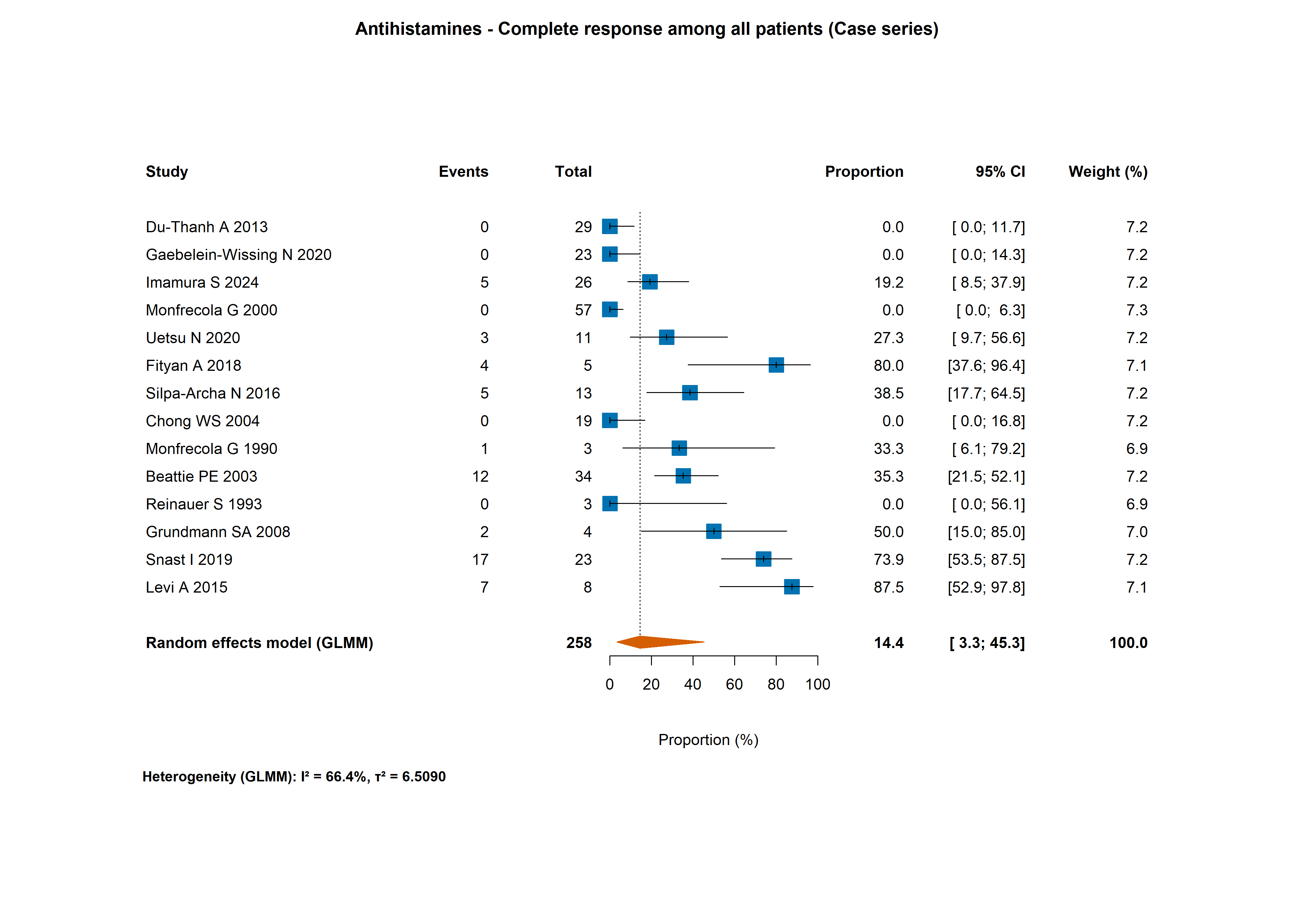

Supplement: Supplementary file 1 [file jcm-14-05736-s001.zip › figS5b.png]

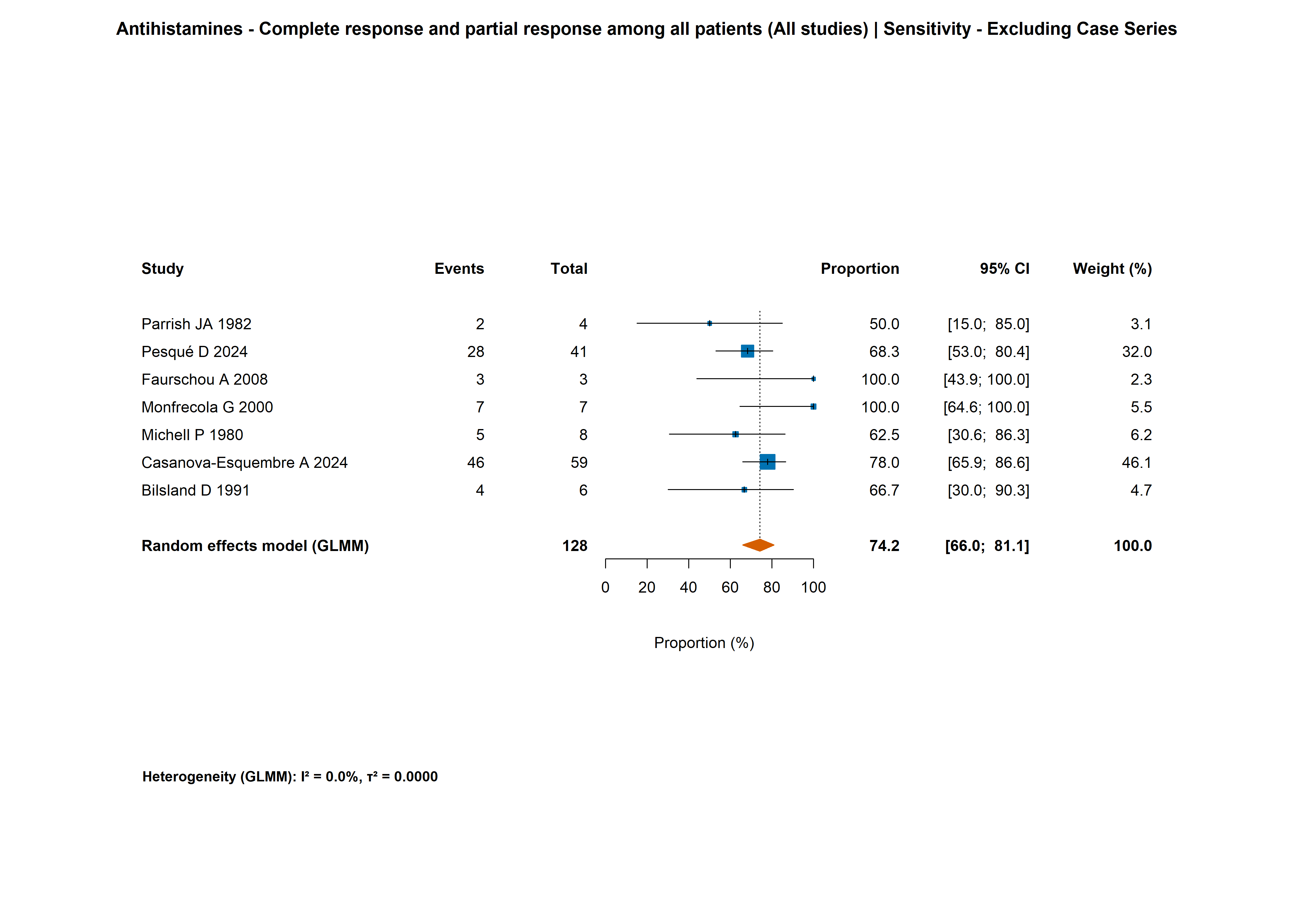

Supplement: Supplementary file 1 [file jcm-14-05736-s001.zip › figS5c.png]

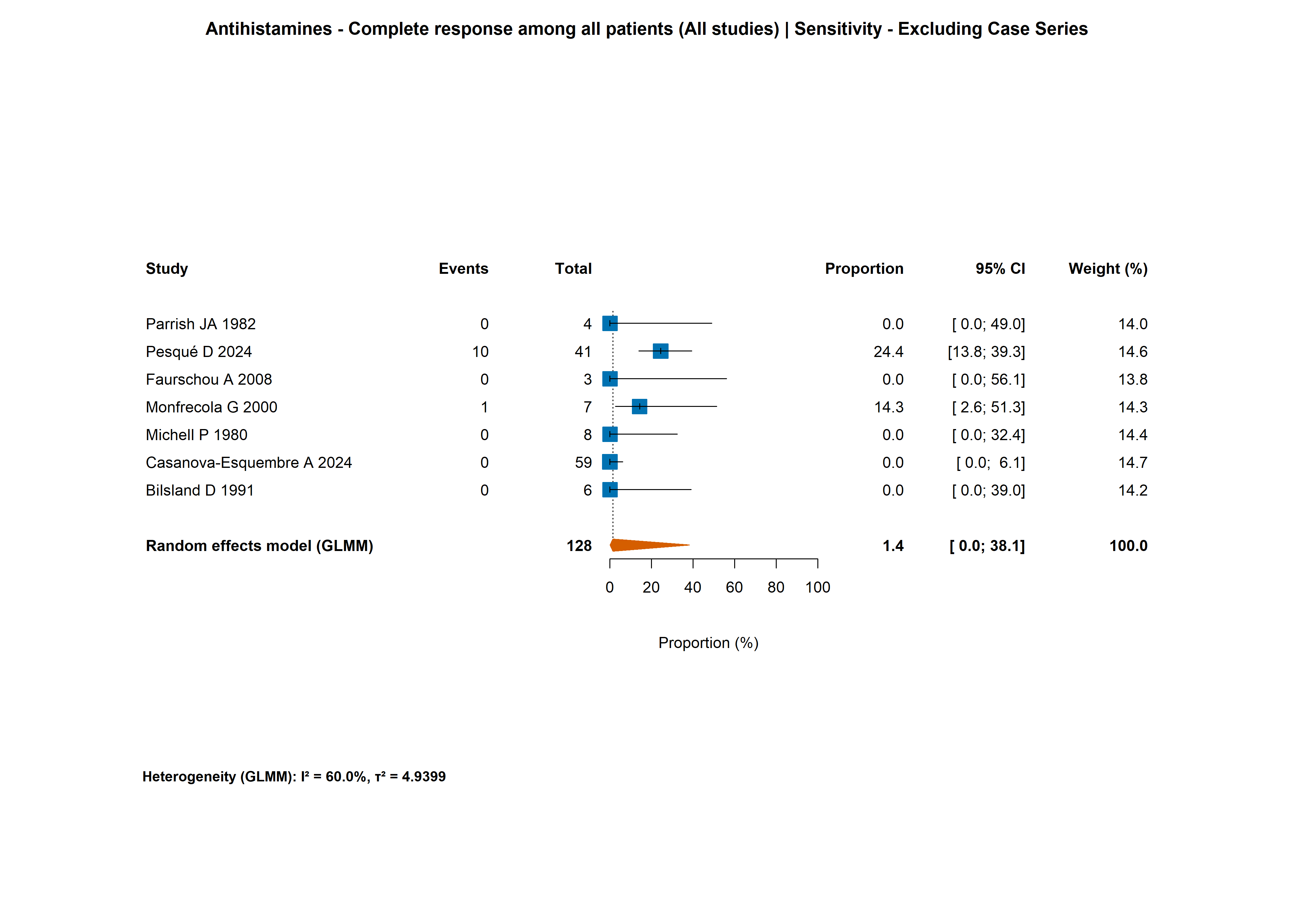

Supplement: Supplementary file 1 [file jcm-14-05736-s001.zip › figS5d.png]

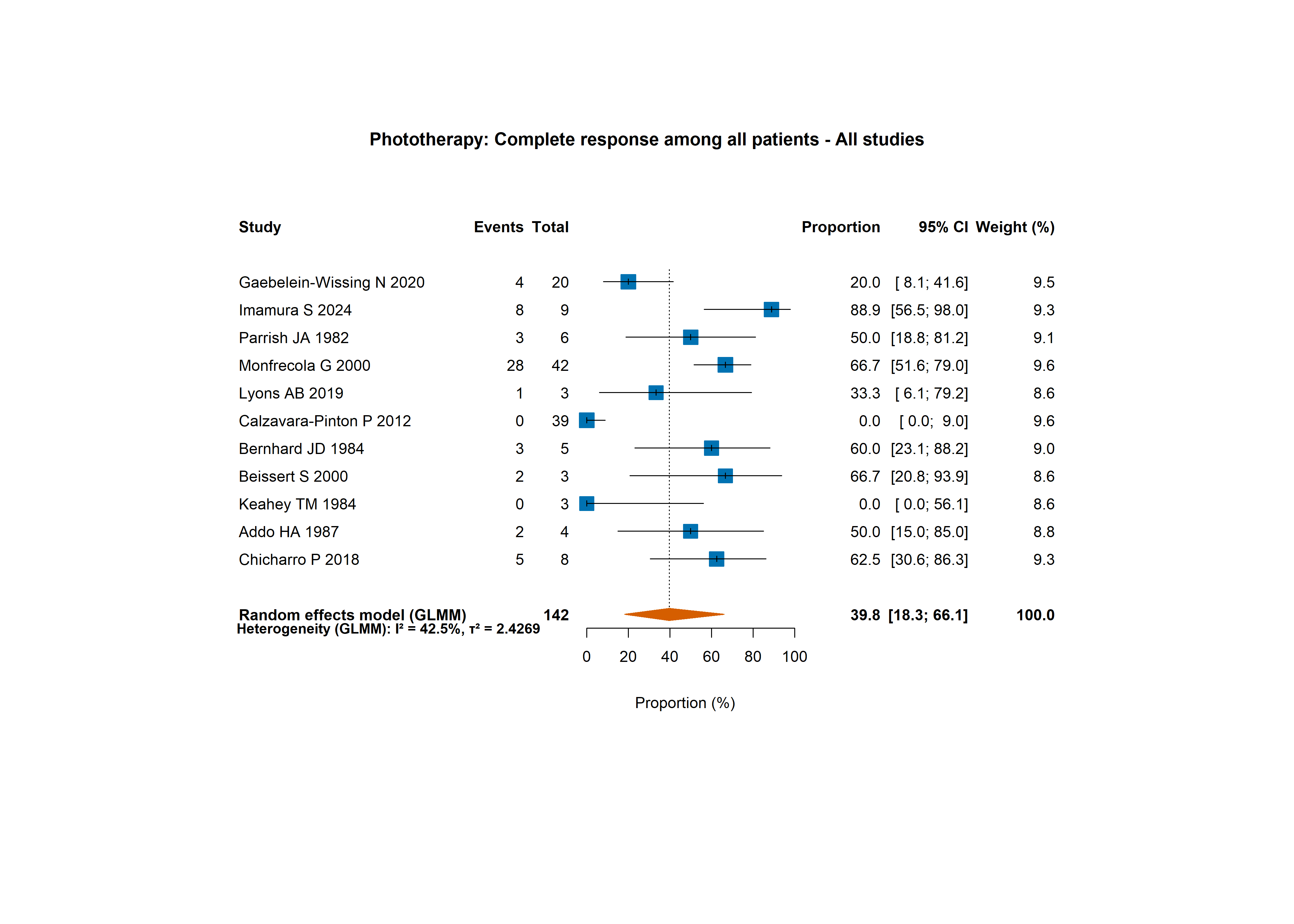

Supplement: Supplementary file 1 [file jcm-14-05736-s001.zip › figS6.png]

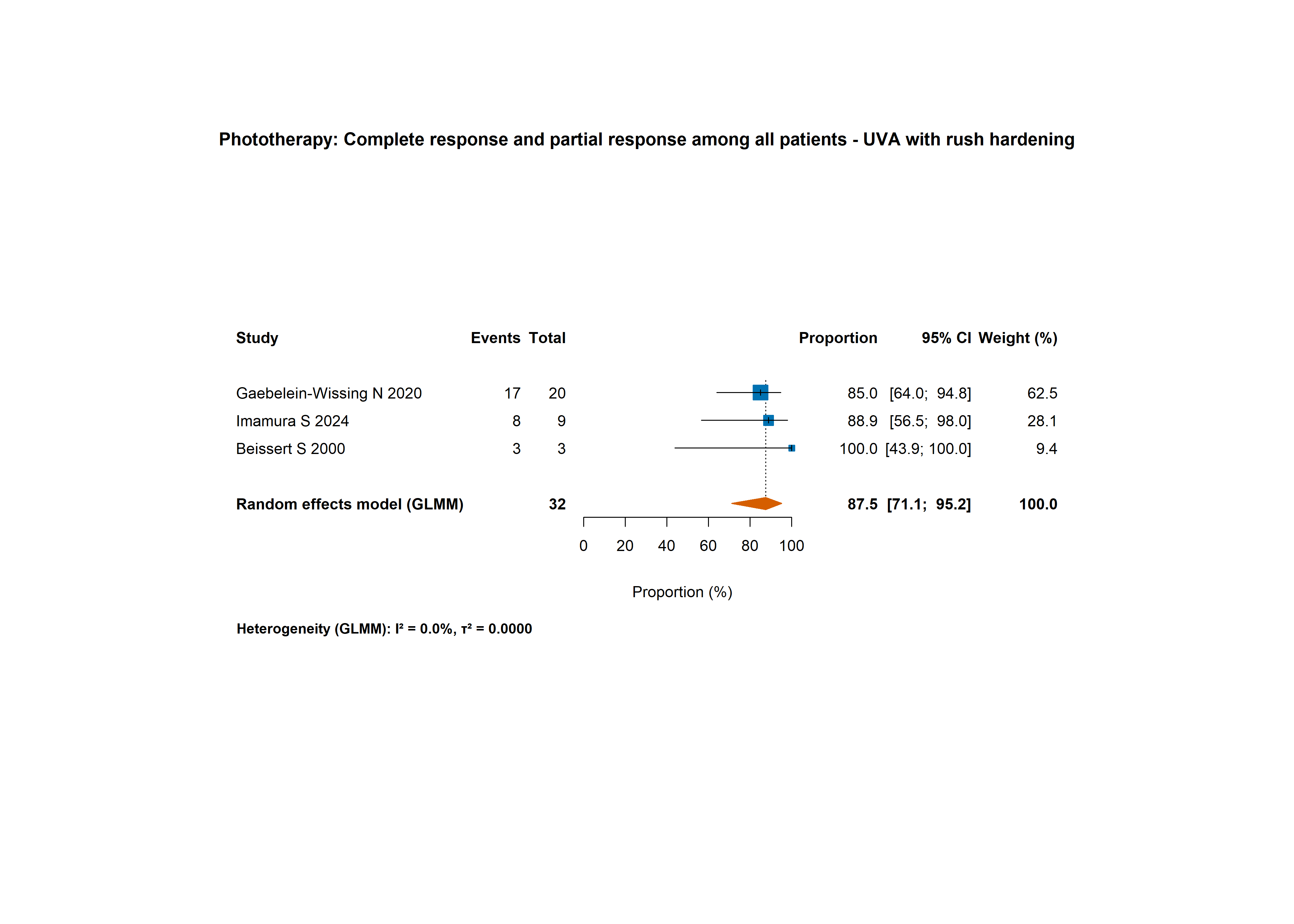

Supplement: Supplementary file 1 [file jcm-14-05736-s001.zip › figS7.png]

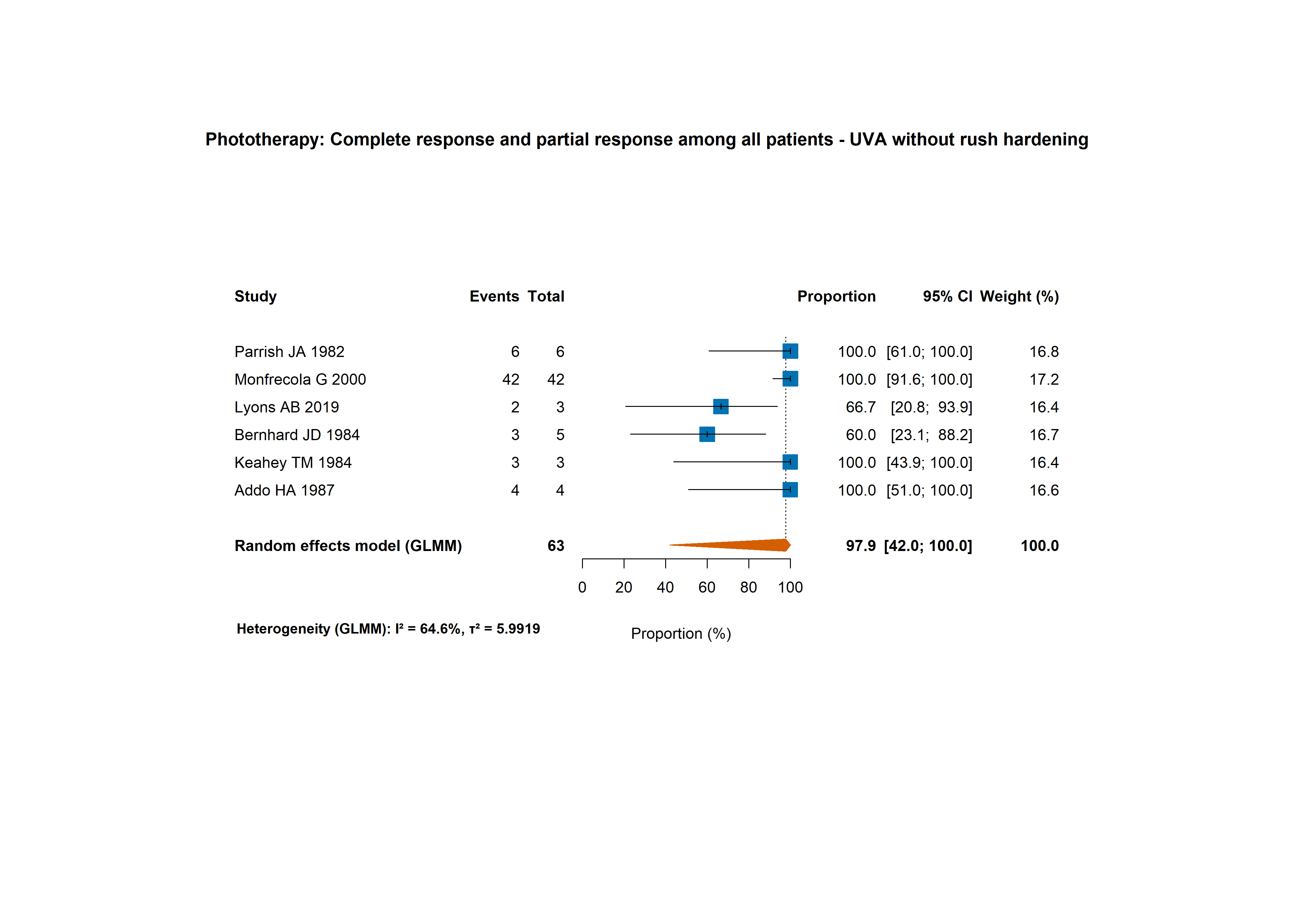

Supplement: Supplementary file 1 [file jcm-14-05736-s001.zip › figS8.png]

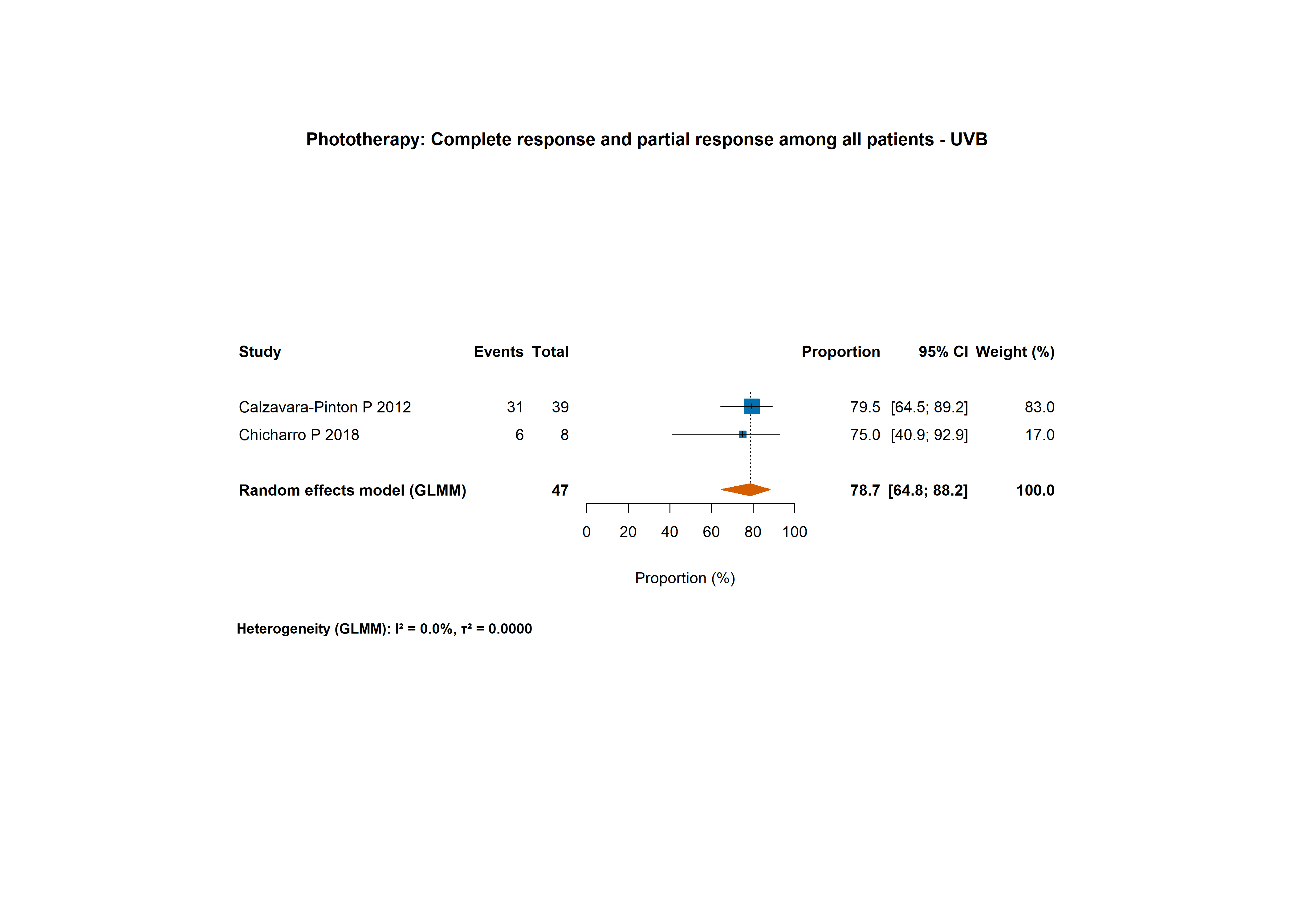

Supplement: Supplementary file 1 [file jcm-14-05736-s001.zip › figS9.png]

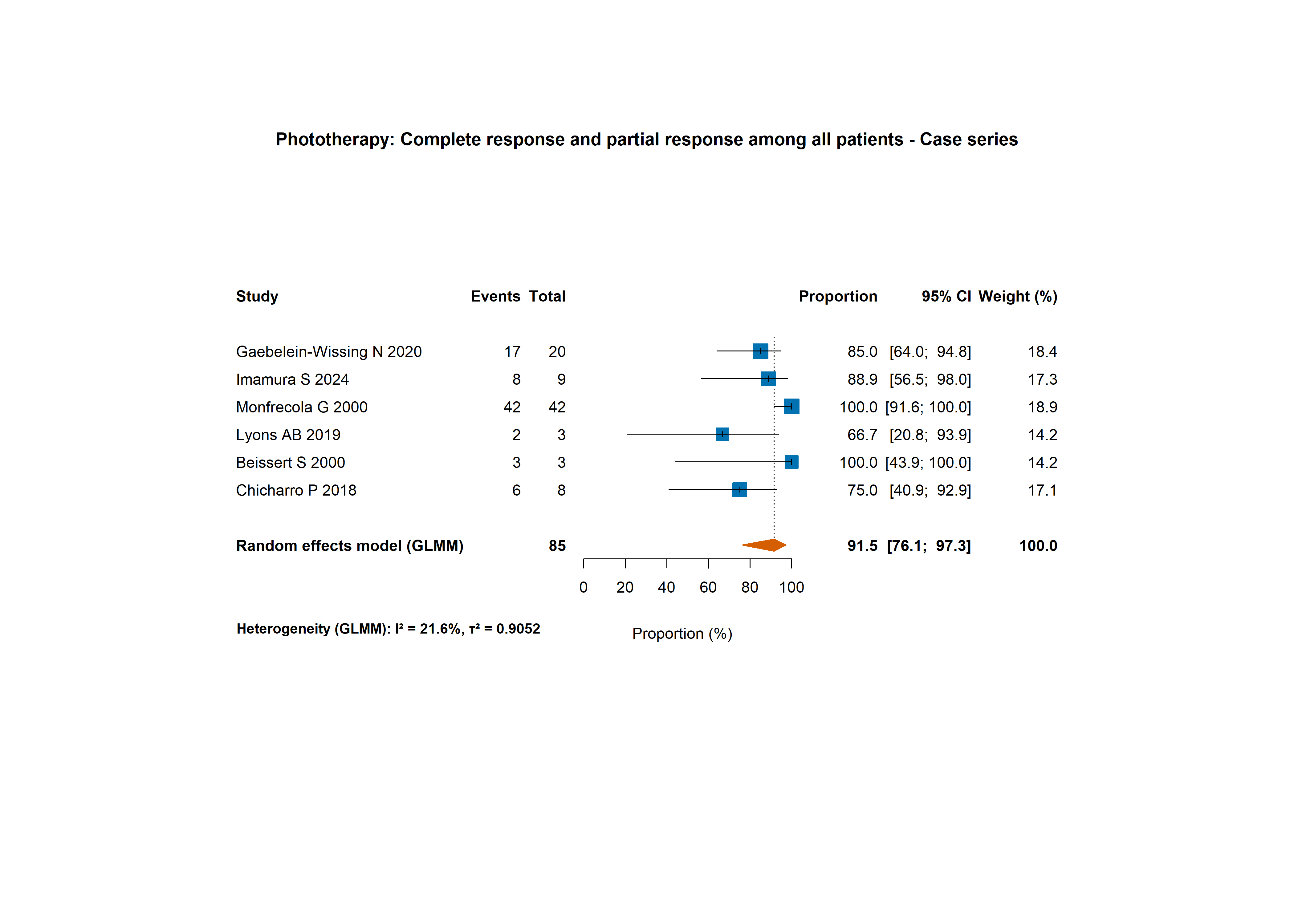

Supplement: Supplementary file 1 [file jcm-14-05736-s001.zip › figS10a.png]

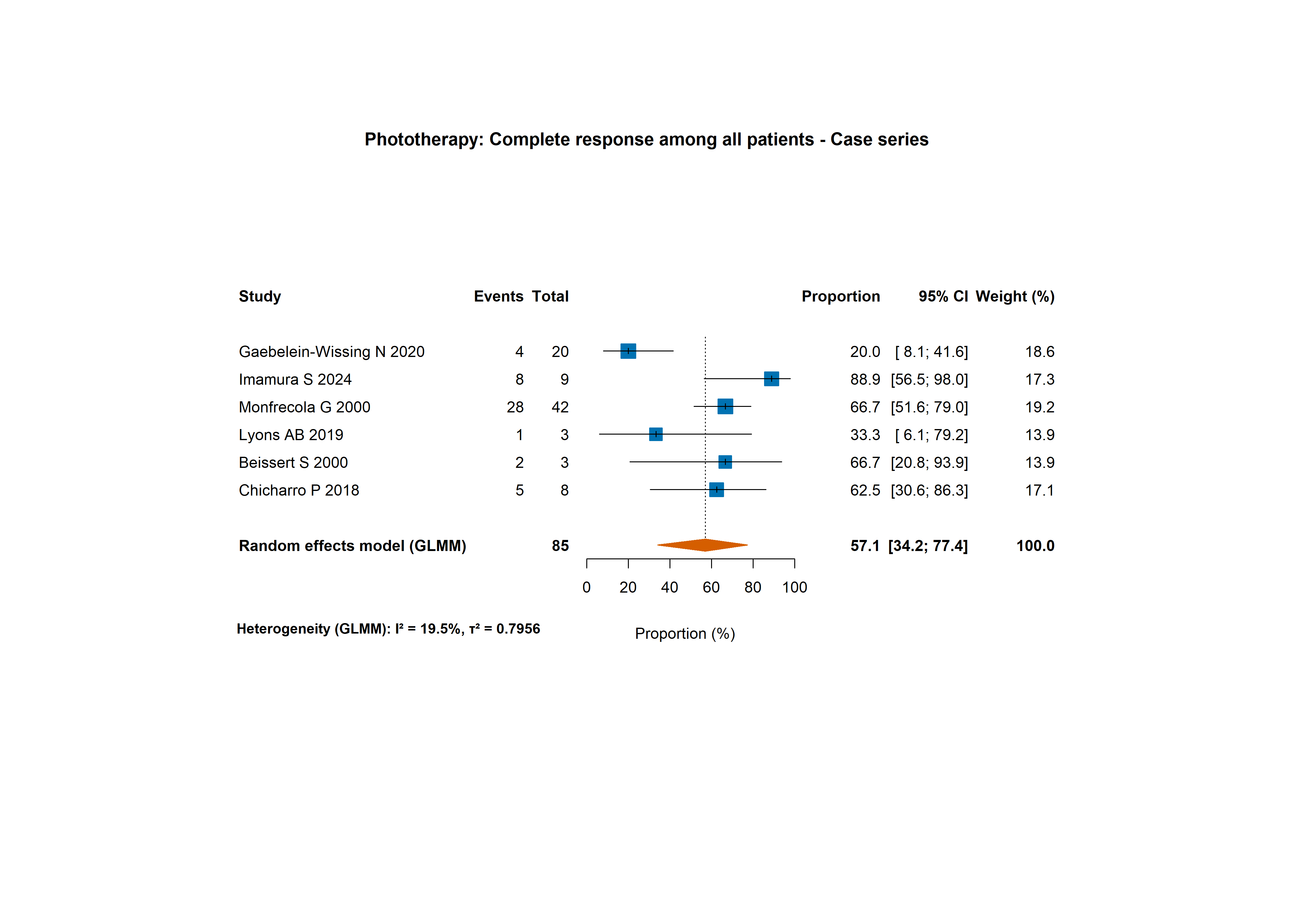

Supplement: Supplementary file 1 [file jcm-14-05736-s001.zip › figS10b.png]

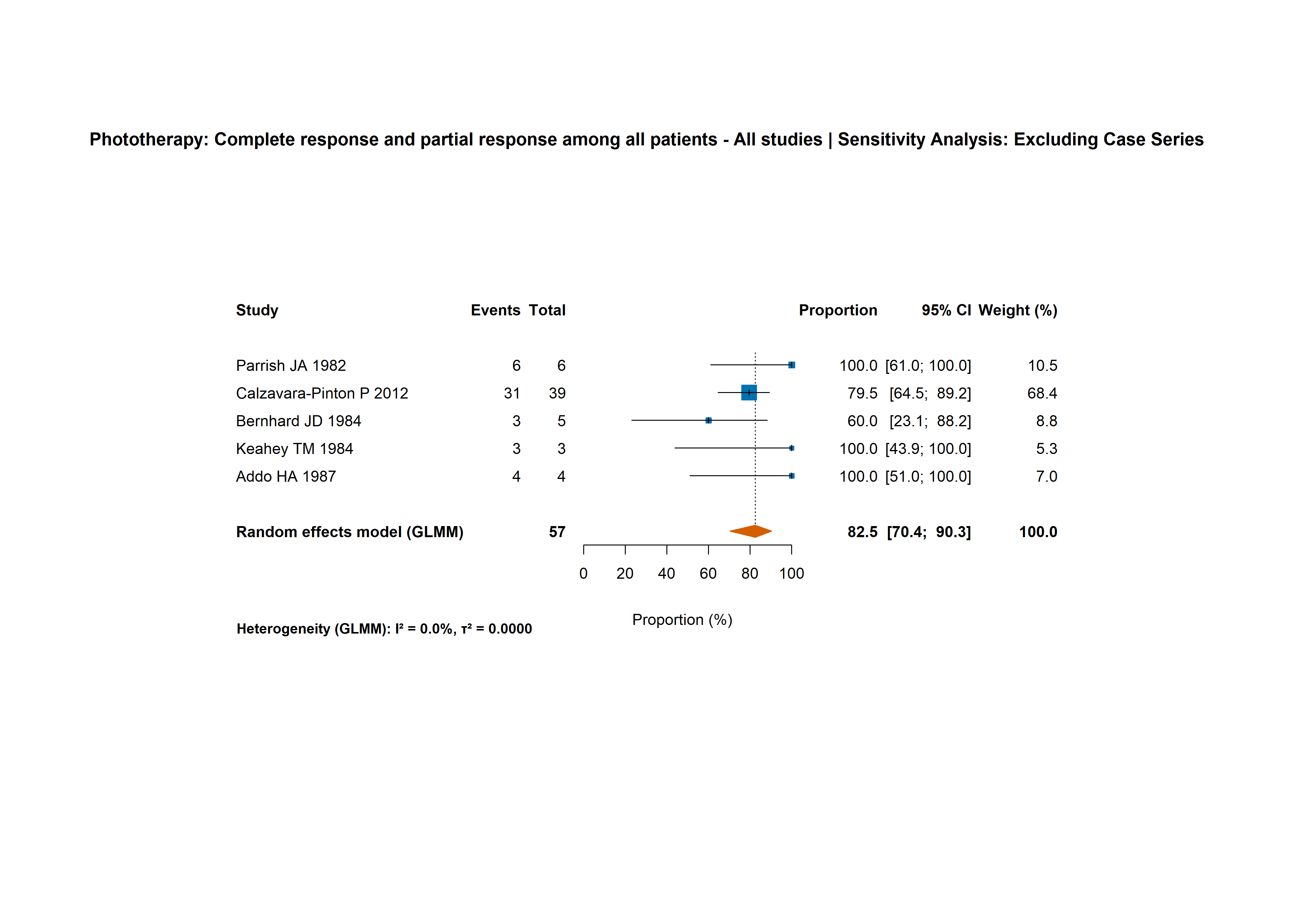

Supplement: Supplementary file 1 [file jcm-14-05736-s001.zip › figS10c.png]

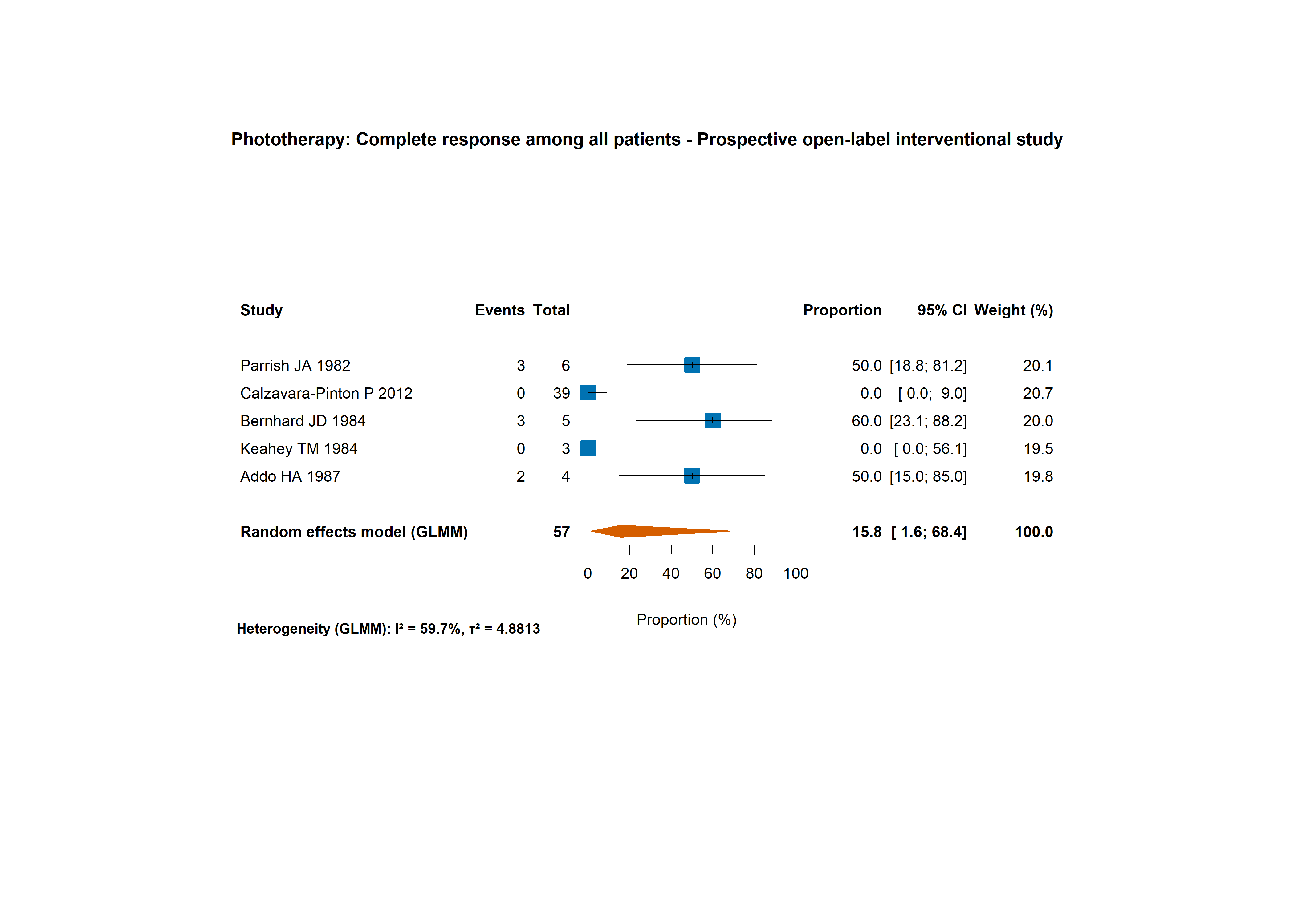

Supplement: Supplementary file 1 [file jcm-14-05736-s001.zip › figS10d.png]

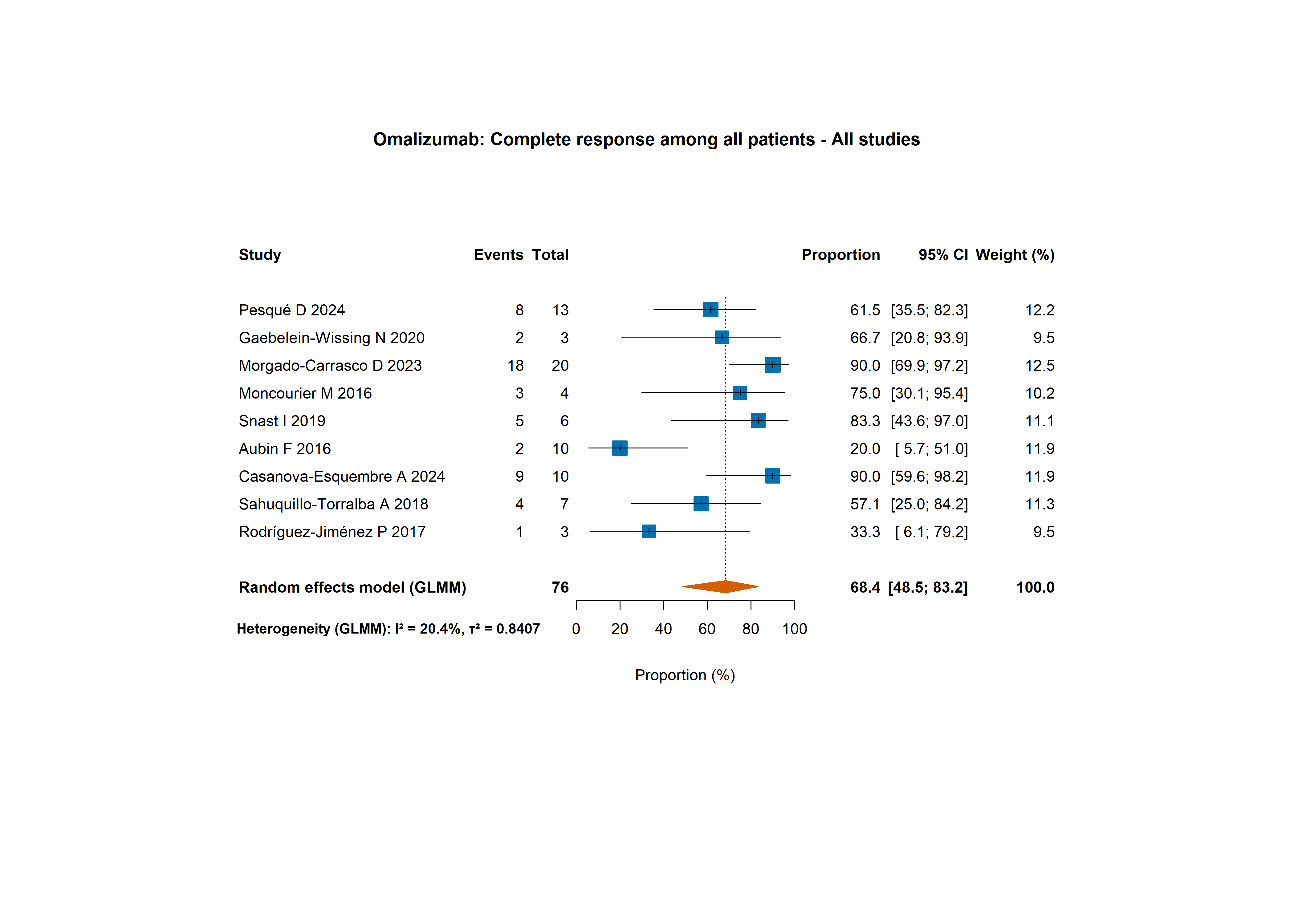

Supplement: Supplementary file 1 [file jcm-14-05736-s001.zip › figS11.png]

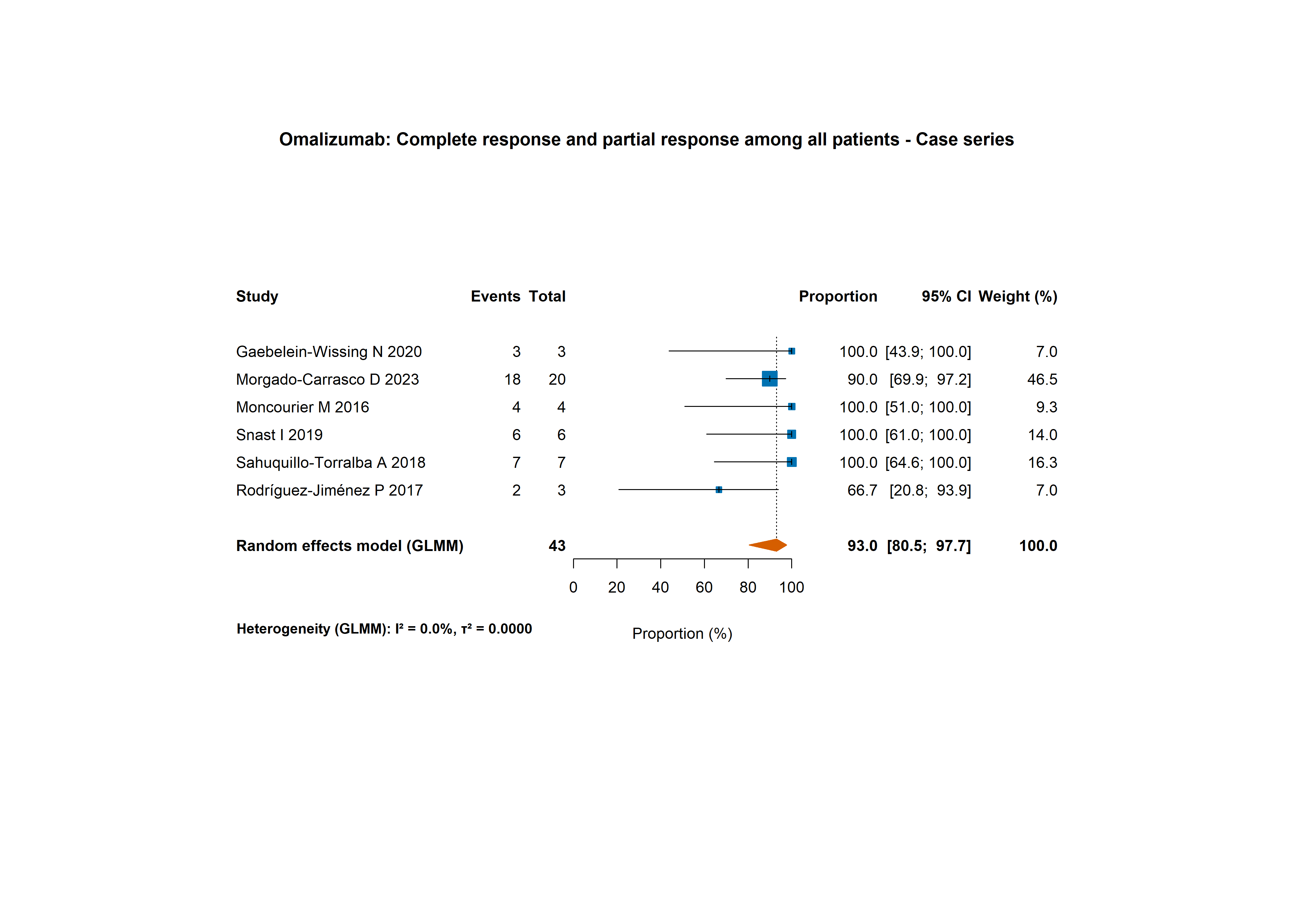

Supplement: Supplementary file 1 [file jcm-14-05736-s001.zip › figS12a.png]

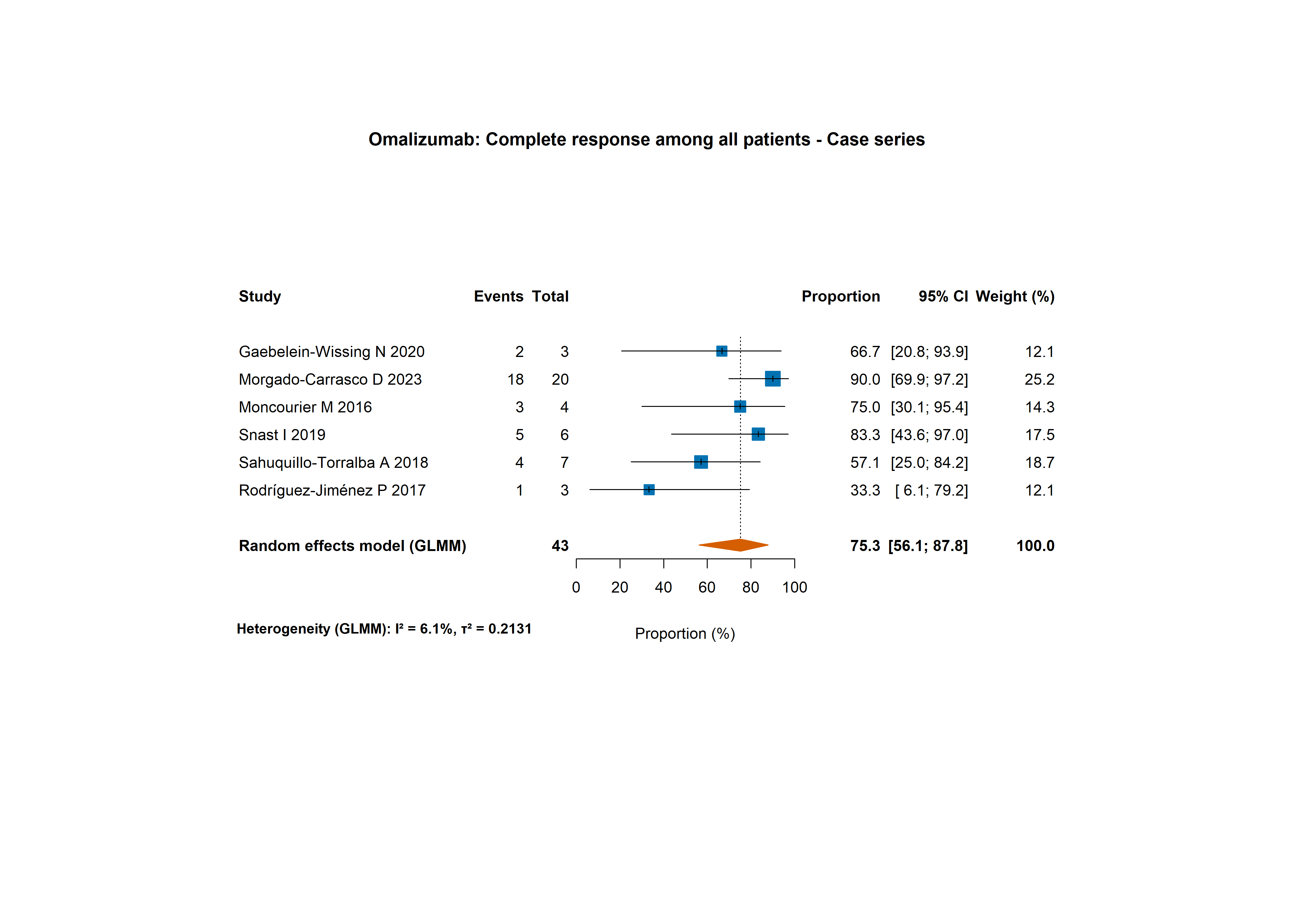

Supplement: Supplementary file 1 [file jcm-14-05736-s001.zip › figS12b.png]

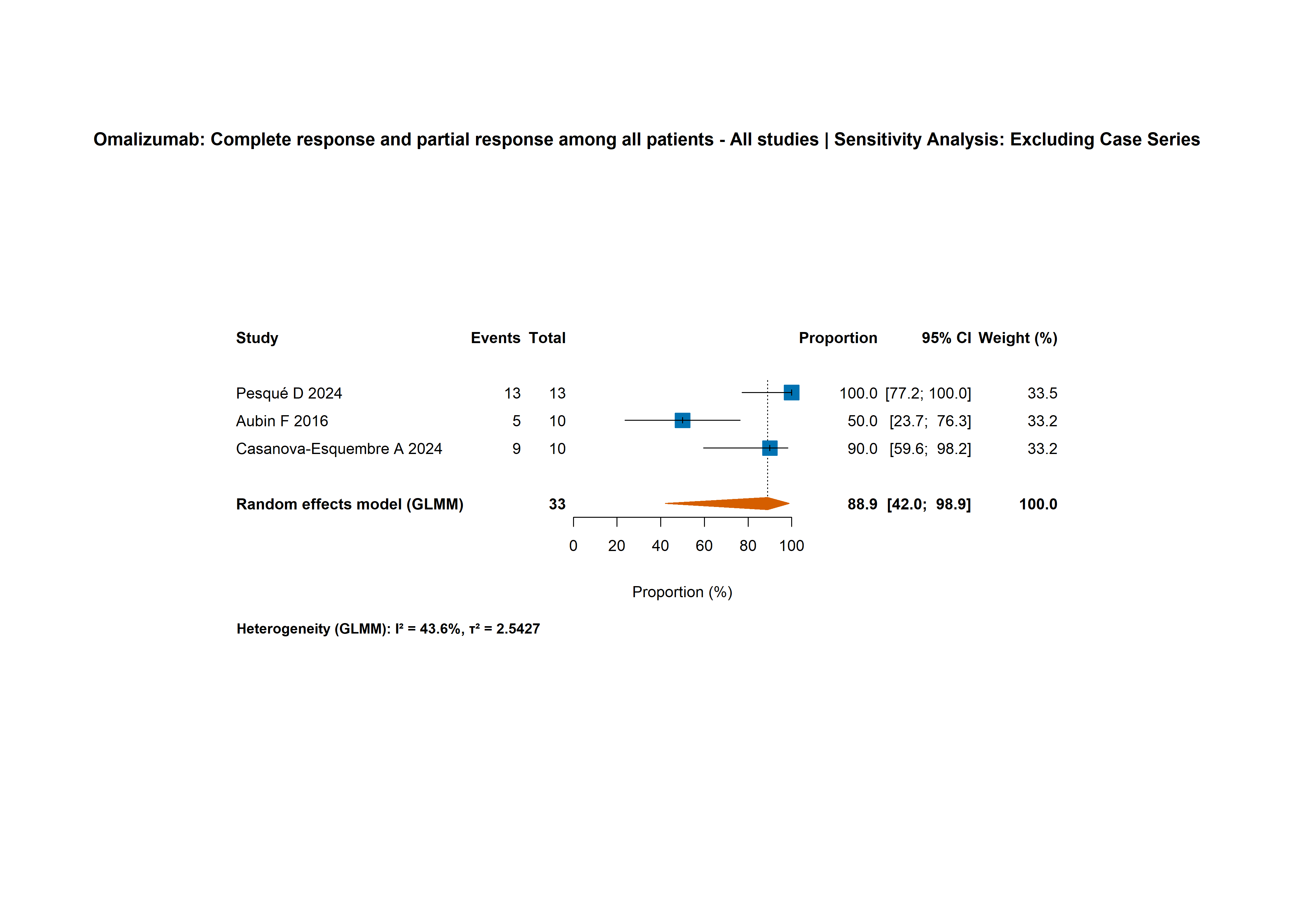

Supplement: Supplementary file 1 [file jcm-14-05736-s001.zip › figS12c.png]

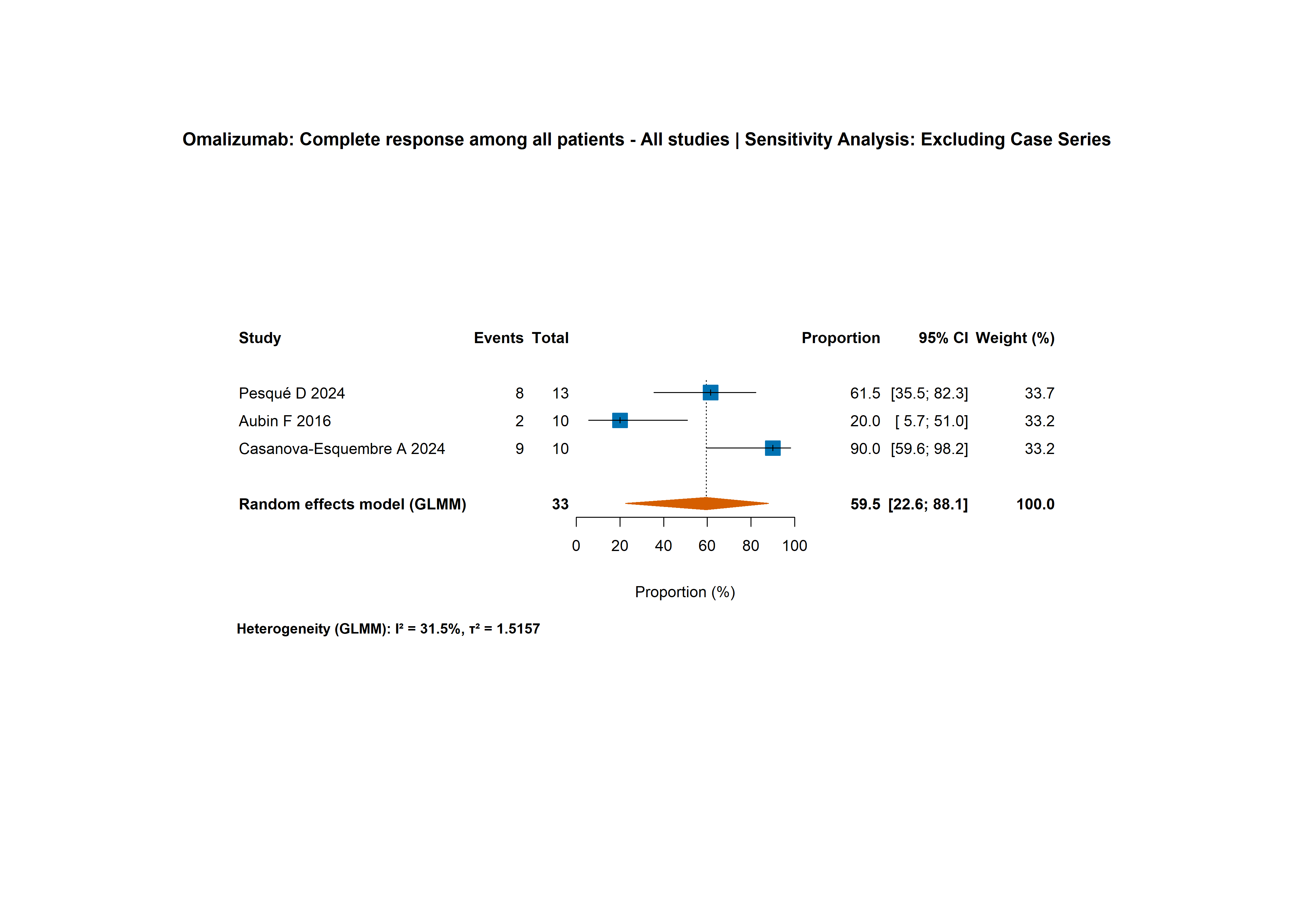

Supplement: Supplementary file 1 [file jcm-14-05736-s001.zip › figS12d.png]
